# ${ SI}$ infection on a dynamic partnership network: characterization of $R_0$

**DOI:** 10.1007/s00285-014-0808-5

**Published:** 2014-07-10

**Authors:** Ka Yin Leung, Mirjam Kretzschmar, Odo Diekmann

**Affiliations:** 1 Mathematical Institute, Utrecht University, Utrecht, The Netherlands; 2 University Medical Centre Utrecht, Utrecht, The Netherlands; 3 Centre for Infectious Disease Control, RIVM, Bilthoven, The Netherlands

**Keywords:** ${ SI}$-infection, Mean field at distance one, Dynamic network, Concurrency, $R_0$, 34D20, 92D30

## Abstract

We model the spread of an ${ SI}$ (*S*usceptible $\rightarrow$ *I*nfectious) sexually transmitted infection on a dynamic homosexual network. The network consists of individuals with a dynamically varying number of partners. There is demographic turnover due to individuals entering the population at a constant rate and leaving the population after an exponentially distributed time. Infection is transmitted in partnerships between susceptible and infected individuals. We assume that the state of an individual in this structured population is specified by its disease status and its numbers of susceptible and infected partners. Therefore the state of an individual changes through partnership dynamics and transmission of infection. We assume that an individual has precisely $n$ ‘sites’ at which a partner can be bound, all of which behave independently from one another as far as forming and dissolving partnerships are concerned. The population level dynamics of partnerships and disease transmission can be described by a set of $(n+1)(n+2)$ differential equations. We characterize the basic reproduction ratio $R_0$ using the next-generation-matrix method. Using the interpretation of $R_0$ we show that we can reduce the number of states-at-infection $n$ to only considering three states-at-infection. This means that the stability analysis of the disease-free steady state of an $(n+1)(n+2)$-dimensional system is reduced to determining the dominant eigenvalue of a $3\times 3$ matrix. We then show that a further reduction to a $2\times 2$ matrix is possible where all matrix entries are in explicit form. This implies that an explicit expression for $R_0$ can be found for every value of $n$.

## Introduction

The role that concurrent partnerships might play in the spread of HIV in sub-Saharan Africa is the subject of an ongoing debate. While simulation studies have shown the large impact that concurrency potentially has on the epidemic growth rate and the endemic prevalence of HIV (Kretzschmar and Morris 1996; Morris and Kretzschmar 1997, 2000; Eaton et al. 2011; Goodreau 2011), the empirical evidence for such a relationship is inconclusive (Lurie and Rosenthal 2010; Reniers and Watkins 2010; Tanser et al. 2011; Kenyon and Colebunders 2012).

Mathematical modelling results have played a key role in fuelling the debate (Watts and May 1992; Kretzschmar and Morris 1996; Morris and Kretzschmar 1997, 2000; Eaton et al. 2011; Goodreau 2011). However, a mathematical framework suitable to derive analytical results is still lacking. At present, simulation studies prevail, and general theory is mainly focused on static networks (Diekmann et al. 1998; Ball and Neal 2008; House and Keeling 2011; Lindquist et al. 2011; Miller et al. 2012; Miller and Volz 2013). This motivated us to develop and analyse a mathematical model for the spread of an ${ SI}$ (*S*usceptible–*I*nfectious) infection along a dynamic network.

In a previous paper (Leung et al. 2012) a model for a dynamic sexual network of a homosexual population is presented that incorporates demographic turnover and allows for individuals to have multiple partners at the same time, with the number of partners varying over time. This network model can be seen as a generalization of the pair formation models (that describe sequentially monogamous populations) to situations where individuals are allowed more than one partner at a time. Pair formation models were first introduced into epidemiology by Dietz and Hadeler (1988) and extended in various ways (Kretzschmar et al. 1994; Inaba 1997; Kretzschmar and Dietz 1998; Xiridou et al. 2003; Heijne et al. 2011; Powers et al. 2011). In the present generalization, individuals have at most $n$ partners at a time. We call $n$ the partnership capacity. In the partnership network individuals are, essentially, collections of $n$ ‘binding sites’ where binding sites can be either ‘free’ or ‘occupied’ (by a partner). In the case that $n=1$ we recover the pair formation model of a monogamous population.

Consider an individual in the sexual network. Since individuals may have several partners simultaneously, the risk of acquiring infection depends on that individual’s partners, but also on their partners, and so on. We would need to keep track of the entire network to fully characterize the risk of infection to an individual. Here we introduce an approximation rather than taking full network information into account: we assume that properties concerning partners of partners can be obtained by averaging over the population. This approximation is termed the ‘mean field at distance one’ assumption (‘mean field at distance one’ should be read as one term; from here on we write this without quotation marks). This assumption relates to what is called ‘effective degree’ in Lindquist et al. (2011), where transmission of infection along a static network is studied (we are, apart from Britton and Lindholm 2010; Britton et al. 2011), not aware of any analytical work so far, on disease transmission across dynamic networks with demography (see e.g. Altmann 1995, 1998; Ferguson and Garnett 2000; Bansal et al. 2010; Kiss et al. 2012; Miller and Volz 2013) and references therein for models incorporating dynamic partnerships in a demographically closed population).

The mean field at distance one assumption is a moment closure approximation obtained by ignoring certain correlations between the states of two individuals that are in a partnership and, as a consequence, this assumption is inconsistent with the assumptions that underlie the partnership network (see e.g. Ferguson and Garnett 2000; Kamp 2010; House and Keeling 2011; Taylor et al. 2012) and references therein for different moment closure approximations on networks). However, this assumption allows us to write down a closed system of ODEs to describe an approximation of the ${ SI}$ infection on the partnership network. If a partnership capacity $n$ is given, then we have an $(n+1)(n+2)$ dimensional system of ODEs.

A large part of the paper is devoted to characterizing the basic reproduction number $R_0$ and proving its threshold character for the nonlinear system of ODEs. This system is quite large already for small $n$. However, by considering only states-at-infection and using the next-generation matrix approach, $R_0$ can be characterized as the dominant eigenvalue of an $n\times n$ matrix. Using the interpretation we can further reduce this and $R_0$ can ultimately be characterized as the dominant eigenvalue of a $2\times 2$ matrix where the entries of this matrix are explicit, and therefore also $R_0$ has an explicit expression. In fact, we are able to interpret $R_0$ in terms of individuals (which are considered in the model specification) and in terms of binding sites.

The structure of the paper is as follows. First, in Sect. 2, we consider the partnership network of Leung et al. (2012) and summarize the main results needed for this paper. Next, in Sect. 3 we superimpose an ${ SI}$-infection on the network and specify the model assumptions. Particular attention is given to the mean field at distance one assumption. The rest of the paper is devoted to characterizing the basic reproduction number $R_0$. For this, in Sect. 4, we first consider the linearisation of the system.

In Sect. 5, which constitutes the core of the paper, we characterize $R_0$ in terms of newly infected binding sites that produce newly infected binding sites. We introduce a transition matrix $\varSigma$ and a transmission matrix $T$ and define $R_0$ as the dominant eigenvalue of the next generation matrix $-T\varSigma ^{-1}$ (Diekmann et al. 2013, Section 7.2). The building blocks for an explicit expression for $R_0$ are presented in Appendix C. We also show that $R_0$ thus defined can be interpreted as the basic reproduction ratio for individuals, since individuals can be considered to be collections of $n$ binding sites. Section 5 can be read independently of the rest of the paper.

The characterization of $R_0$ in Sect. 5 does not, by itself, provide a mathematical proof that the disease-free steady state is stable for $R_0<1$ and unstable for $R_0>1$. We provide such a proof in Sect. 6. The proof is based on the Perron–Frobenius theory of spectral properties of positive and positive-off-diagonal irreducible matrices. In particular we use that

- ${{\mathrm{sign}}}(R_0- 1) = {{\mathrm{sign}}}(r)$ where $r$ is the Malthusian parameter (i.e. the dominant eigenvalue) of the matrix $T+ \varSigma$
- the linearised system derived in Sect. 4 can be mapped in a natural way to the binding-site system defined by the matrices $\varSigma$ and $T$, while preserving positivity.

The final Sect. 7 provides conclusions and plans for future work. Some more technical calculations are left for the six appendices. In particular, in Appendix B we show with explicit calculations for the case $n=2$ (suggested to us by Pieter Trapman (personal communication, 26 August, 2013)) that states of partners are not independent of one another, implying that the mean field at distance one assumption yields only an approximate and not an exact description.

## The partnership network

In this section we will give a summary of the specification of the partnership network and of the main results presented in Leung et al. (2012).

Consider a population of homosexual individuals—all with partnership capacity $n$. The partnership capacity is the maximum number of simultaneous partners an individual may have. One may think of an individual as having $n$ binding sites. Binding sites are either ‘occupied’ (by a partner) or ‘free’. We assume that *binding sites of an individual behave independently from one another* as far as forming and dissolving partnerships are concerned. Furthermore, individuals enter (‘birth’) and leave (‘death’) the sexually active population.

The model specification begins at the individual level. The state of an individual is given by $k$, the number of occupied binding sites, $k=0,\ldots ,n$. Consider one individual born at time $t_b$ and suppose it does not die in the time interval under consideration. An occupied binding site becomes free at rate $\sigma +\mu$, where $\sigma$ corresponds to ‘separation’ and $\mu$ to ‘death of partner’. A free binding site becomes occupied at rate $\rho F$, where $F$ denotes the fraction of free binding sites in the pool of all binding sites in the population. The possible state transitions and the rates at which they occur are:

$$\begin{aligned} k\rightarrow k+1&\qquad \text { with rate } \rho (n-k)F,\\ k\rightarrow k-1&\qquad \text { with rate } (\sigma +\mu )k. \end{aligned}$$

The probability that an individual is in state $k$ at age $a$ is denoted by $p_k(t_b,a)$, where $t_b$ denotes the time of birth. A newborn individual has $n$ free binding sites, i.e.

$$\begin{aligned} p(t_b,0)=\begin{pmatrix}1\\ 0\\ \vdots \\ 0\end{pmatrix}\!. \end{aligned}$$

Let $A=A(F)$ denote the matrix corresponding to the state transitions described above. So, as an example, for $n=2$, the matrix $A$ is as follows:

$$\begin{aligned} A=\begin{pmatrix}-2\rho F&{}\quad \sigma +\mu &{}\quad 0\\ 2\rho F&{}\quad -(\rho F+\sigma +\mu )&{}\quad 2(\sigma +\mu )\\ 0&{}\quad \rho F&{}\quad -2(\sigma +\mu )\end{pmatrix}\!. \end{aligned}$$

Note that, throughout this paper, we will use the convention that, for a transition matrix $M=(m_{ij})$, $m_{ij}$ denotes the probability per unit of time at which a transition from $j$ to $i$ is made (instead of the transition from $i$ to $j$, as it is common in the stochastic community.)

Then, as long as the individual does not die, we have

$$\begin{aligned} \frac{\partial p}{\partial a}(t_b,a)&=A(F(t_b+a))p(t_b,a). \end{aligned}$$

We assume a stationary age distribution which is exponential with parameter $\mu$, so it has probability density function

1 $$\begin{aligned} a\mapsto \mu e^{-\mu a}. \end{aligned}$$

Then, in a deterministic description of a large population, the fraction of the population in state $k$ at time $t$ is

2 $$\begin{aligned} P_k(t)&=\int _0^\infty \mu e^{-\mu a}p_k(t-a,a)da=\int _{-\infty }^t\mu e^{-\mu (t-\alpha )}p_k(\alpha ,t-\alpha )d\alpha . \end{aligned}$$

The fraction of free binding sites $F$ is defined as

3 $$\begin{aligned} F(t)=\frac{1}{n}\sum _{k=0}^n(n-k)P_k(t). \end{aligned}$$

Due to the assumption of independence of binding sites with respect to partnership dynamics, the dynamics of $F$ decouple as stated in Lemma 1 below (the proof is presented in Leung et al. 2012).

### **Lemma 1**

The fraction of free binding sites $F$ satisfies the differential equation

4 $$\begin{aligned} \frac{dF}{dt}=\mu +(\sigma +\mu )(1-F)-\rho F^2-\mu F. \end{aligned}$$

Consequently,

$$\begin{aligned} F(t)\rightarrow \bar{F}, \end{aligned}$$

for $$t\rightarrow \infty $$, where

5 $$\begin{aligned} \bar{F}=\frac{\sqrt{(\sigma +2\mu )(4\rho +\sigma +2\mu )}-(\sigma +2\mu )}{2\rho }. \end{aligned}$$

This convergence to $\bar{F}$ motivates us to take $F$ constant and equal to $\bar{F}$ (note, incidentally, that $\bar{F}$ does not depend on the partnership capacity $n$). As a consequence the argument $t_b$ in $p_k(t_b,a)$ no longer matters and $P_k(t)=P_k$ is independent of time. In fact, one finds that

$$\begin{aligned} P_k=\left( \begin{array}{c}n\\ k\end{array}\right) \int _0^\infty \mu e^{-\mu a}\epsilon (a)^k(1-\epsilon (a))^{n-k}da, \end{aligned}$$

where

$$\begin{aligned} \epsilon (a)=\frac{\rho \bar{F}}{\rho \bar{F}+\sigma +\mu }(1-e^{-(\rho \bar{F}+\sigma +\mu )a}) \end{aligned}$$

is the probability that a binding site is occupied at age $a$, given that the ‘owner’ of the binding site is alive. . We can get rid of the integral by using the binomium of Newton to expand $\epsilon (a)^k(1-\epsilon (a))^{n-k}$ and compute the integral of an exponential function:

6 $$\begin{aligned} P_k=&\left( {\begin{array}{c}n\\ k\end{array}}\right) \mu \left( \frac{\rho \bar{F}}{\rho \bar{F}+\sigma +\mu }\right) ^{n}\nonumber \\&\sum _{j=0}^{n-k}\sum _{i=0}^k\left( {\begin{array}{c}n-k\\ j\end{array}}\right) \left( {\begin{array}{c}k\\ i\end{array}}\right) (-1)^i\left( \frac{\sigma +\mu }{\rho \bar{F}}\right) ^j\frac{1}{\mu +(\rho \bar{F}+\sigma +\mu )(n-k-j+i)}. \end{aligned}$$

So we have explicit expressions for the degree distribution $P=(P_k)_{k=0}^n$.

There are two probability distributions that play a more important role in the characterization of $R_0$. First, consider an individual that acquires a new partner. We assume, in accordance with (3), that this newly acquired partner will have state $k$ with probability

7 $$\begin{aligned} q_{k}=\frac{(n-k+1)P_{k-1}}{\sum _{l=0}^n(n-l)P_l}=\frac{(n-k+1)P_{k-1}}{n\bar{F}}. \end{aligned}$$

(A potential partner with state $k-1$ has $(n-k+1)$ free binding sites. Immediately after a match is made it will have state $k$. The denominator serves to renormalise into a probability distribution.) This assumption gives us information on the state of an individual in a randomly chosen partnership, as expressed in the next lemma.

### **Lemma 2**

Choose an individual by first sampling a partnership from the pool of all partnerships and next choosing one of the two partners at random. The probability that this individual has $k$ partners equals

8 $$\begin{aligned} Q_k=\frac{kP_k}{\sum _{l=1}^nlP_l}=\frac{kP_k}{n(1-\bar{F})}. \end{aligned}$$

Note that Lemma 2 does *not* imply that the states of the two individuals in this partnership are independent of one another. Indeed, they are not. Information about the number of partners of one of the individuals provides some information about the duration of the partnership and thus influences the probability that the other individual has $k$ partners (or, in other words, there exists degree correlation in this network); see Appendix B for explicit calculations for $n=2$. (We have, so far, not calculated degree correlations for general $n$.)

Note that the model specification is deterministic in the sense that it concerns expected values for a population of infinite size. Partnership formation is at random between two free binding sites. As a consequence of mass action and infinite population size, partnership formation with oneself or multiple partnerships with the same individual occur with probability zero. For the same reason clustering does not occur in the network. It should be possible to formulate a stochastic version for a population of size $N$ and derive the present description by considering the limit $N\rightarrow \infty$. We conjecture that all the previous statements hold in the limit. In particular clustering disappears in the limit, i.e. the probability that a path of a fixed finite length contains a loop goes to zero in the limit.

Finally, to summarize, we have three degree distributions, i.e. probability distributions for the number of partners of an individual, that we will use throughout this paper:

- $P=(P_k)$ for a random individual,
- $q=(q_k)$ for an individual who just acquired a partner (but is otherwise randomly chosen),
- $Q=(Q_k)$ for an individual in a randomly chosen partnership.

## Superimposing transmission of an infectious disease

We consider an ${ SI}$ infection spreading on the dynamic sexual network described in Sect. 2. We assume that individuals become infectious at the very instant that they become infected and stay infectious (with the same infectiousness) for the rest of their life.

### i-states and i-dynamics

The model specification begins at the i-level (i for individual). We classify individuals as either susceptible (indicated by the symbol $-$) or infectious (indicated by $+$). We assume that the $\pm$ classification has no influence whatsoever on partnership formation and separation nor on the probability per unit of time of dying.

The state of an individual is now a triple $(x, k_-,k_+)$, where $x$ is either $+$ or $-$ and $k_-$ and $k_+$ are nonnegative integers with $0\le k_- +k_+\le n$. The $x$ specifies whether the individual itself is susceptible or infectious, $k_{-}$ specifies the number of its susceptible partners, and $k_+$ specifies the number of its infectious partners.

#### Demographic change of i-states

Consider an individual and suppose it does not die in the period under consideration. There are two types of state transitions: those that contribute to demography and those that involve transmission of infection.

We let $F_-$ denote the fraction of the total pool of binding sites that is free and belongs to a susceptible individual and let $F_+$ denote the fraction that is free and belongs to an infectious individual so $F_-+F_+=\bar{F}$. We shall say that a binding site is susceptible or infectious if the ‘owner’ is so.

The possible state transitions and corresponding rates that involve partnership formation, separation, and death of a partner are as follows:


#### Transmission (mean field at distance one)

Next, consider the transmission events. A susceptible having a binding site that is occupied by an infectious partner, gets infected by this partner at rate $\beta$. There is more than one way in which transmission events show up as i-level state transitions. First of all, we have the possibility that a susceptible individual $u$ gets infected by one of its infectious partners. This occurs at rate $\beta$ times the number of infectious partners $u$ has, i.e.,

$$\begin{aligned} (-,k_-,k_+)\rightarrow (+,k_-,k_+) \quad \qquad \qquad \text { with rate }\beta k_+. \end{aligned}$$

Here we have assumed that the frequency of sex acts within one partnership does not depend on concurrent other partnerships.

It is also possible that a partner $v$ of $u$ (with $u$ either susceptible or infectious) becomes infected by one of $v$’s infectious partners (which includes $u$ if $u$ is infectious). Of course the probability that this happens depends on the actual configuration in terms of number of partners of $v$ and their infection status. That information is, however, not incorporated in our description.

Therefore, we assume that we can average over all possibilities (we call this ‘mean field at distance one’). This assumption is an approximation that we make in order to close the infectious disease model within our limited bookkeeping framework; we will come back to this in more detail in Sect. 3.2.2. More concretely we assume that rates $\varLambda _{\pm }(t)$ exist such that

9 $$\begin{aligned} (-,k_-,k_+)&\rightarrow (-,k_{-}-1,k_++1)\qquad \text { with rate }\beta \varLambda _-(t)k_-,\nonumber \\ (+,k_-,k_+)&\rightarrow (+,k_{-}-1,k_++1)\qquad \text { with rate }\beta \varLambda _+(t)k_-, \end{aligned}$$

and that we can specify $\varLambda _{\pm }(t)$ as appropriate population averages. But before we can provide this specification in Sect. 3.2.2, we have to define the relevant population-level quantities. For this we need to first consider the i-level dynamics.

#### i-level dynamics

We have now described all i-states and the possible changes in i-states. The i-level dynamics are as follows. Newborn individuals are in state $(-,0,0)$ (we call this the i-state-at-birth), i.e. at birth an individual is susceptible and has no partner at all. Let $p_\ell (t_b,a)$ denote the probability that an individual born at time $t_b$ is in state $\ell$ at age $a$ given that the individual does not die in the period under consideration, where $\ell$ is any allowed triple $(\pm ,k_-,k_+)$. By choosing a way to order the $\ell$’s, we can think of $p$ as a vector. This ordering then also allows us to construct a matrix

$$\begin{aligned} B=B(F_\pm , \varLambda _\pm ) \end{aligned}$$

on the basis of the transition rates that are described in Sects. 3.1.1 and 3.1.2.

Then the matrix $B$ allows us to describe the dynamics of $p$. As long as the individual does not die,

10 $$\begin{aligned} \frac{\partial p}{\partial a}(t_b,a)=B\left( F_\pm (t_b+a), \varLambda _\pm (t_b+a)\right) \ p(t_b,a), \end{aligned}$$

with

11 $$\begin{aligned} p(t_b,0)=\left( \begin{array}{c}1\\ 0\\ \vdots \\ 0\end{array}\right) \end{aligned}$$

if $(-,0,0)$ is chosen as the first triple in our list.

Finally, as an example, we write out the matrix $B$ for $n=2$. If we order the twelve states $(\pm ,k_-,k_+)$ as $(-,0,0)$, $(-,1,0)$, $(-,2,0)$, $(-,0,1)$, $(-,1,1)$, $(-,0,2)$, $(+,0,0)$, $(+,1,0)$, $(+,2,0)$, $(+,0,1)$, $(+,1,1)$, $(+,0,2)$, then $B$ is of the form

$$\begin{aligned} B=\begin{pmatrix} B_1 &{}\quad 0\\ B_2 &{}\quad B_3 \end{pmatrix}, \end{aligned}$$

with the $B_i$ being $6\times 6$ matrices. $B_1$ describes the transitions between $-$ states:

$$\begin{aligned} B_1=\begin{pmatrix} (B_1)_{11} &{}\quad \sigma +\mu &{}\quad 0 &{}\quad \sigma +\mu &{}\quad 0 &{}\quad 0 \\ 2\rho F_- &{}\quad (B_1)_{22} &{}\quad 2(\sigma +\mu ) &{}\quad 0 &{}\quad \sigma +\mu &{}\quad 0 \\ 0 &{}\quad \rho F_- &{}\quad (B_1)_{33} &{}\quad 0 &{}\quad 0 &{}\quad 0\\ 2\rho F_+ &{}\quad \beta \varLambda _- &{}\quad 0 &{}\quad (B_1)_{44} &{} \quad \sigma +\mu &{}\quad 2(\sigma +\mu )\\ 0&{}\quad \rho F_+ &{}\quad 2\beta \varLambda _-&{}\quad \rho F_-&{}\quad (B_1)_{55}&{}\quad 0\\ 0&{}\quad 0&{}\quad 0&{}\quad \rho F_+&{}\quad \beta \varLambda _-&{}\quad (B_1)_{66} \end{pmatrix}\!, \end{aligned}$$

with $(B_1)_{jj}=-\sum _{i=1}^6((B_1)_{ij}+(B_2)_{ij})$, and where $B_2$ describes the transitions from $-$ to $+$ states:

$$\begin{aligned} B_2=\begin{pmatrix} 0 &{}\quad 0 &{}\quad 0 &{}\quad 0 &{}\quad 0 &{} \quad 0\\ 0 &{}\quad 0 &{}\quad 0 &{} \quad 0 &{} \quad 0 &{} \quad 0\\ 0 &{}\quad 0 &{}\quad 0 &{}\quad 0 &{} \quad 0 &{}\quad 0\\ 0 &{}\quad 0 &{}\quad 0 &{}\quad \beta &{}\quad 0 &{}\quad 0\\ 0 &{}\quad 0 &{}\quad 0 &{} \quad 0 &{}\quad \beta &{}\quad 0\\ 0 &{}\quad 0 &{}\quad 0 &{}\quad 0 &{}\quad 0 &{}\quad 2\beta \\ \end{pmatrix}\!, \end{aligned}$$

and $B_3$ describes the transitions between $+$ states:

$$\begin{aligned} B_3=\begin{pmatrix} (B_3)_{11} &{}\quad \sigma +\mu &{}\quad 0 &{} \quad \sigma +\mu &{}\quad 0 &{} \quad 0\\ 2\rho F_- &{}\quad (B_3)_{22} &{}\quad 2(\sigma +\mu ) &{}\quad 0 &{}\quad \sigma +\mu &{}\quad 0\\ 0 &{} \rho F_- &{}\quad (B_3)_{33} &{}\quad 0 &{}\quad 0 &{}\quad 0\\ 2\rho F_+ &{} \quad \beta \varLambda _+ &{}\quad 0 &{}\quad (B_3)_{44} &{}\quad \sigma +\mu &{}\quad 2(\sigma +\mu )\\ 0 &{}\quad \rho F_+ &{}\quad 2\beta \varLambda _+ &{}\quad \rho F_- &{}\quad (B_3)_{55} &{}\quad 0\\ 0 &{}\quad 0 &{}\quad 0 &{}\quad \rho F_+ &{}\quad \beta \varLambda _+ &{}\quad (B_3)_{66}\\ \end{pmatrix}\!, \end{aligned}$$

with $(B_3)_{jj}=-\sum _{i=1}^6(B_3)_{ij}$. So in this way, one can construct the matrix $B$ explicitly.

### Bookkeeping on the p-level and feedback

We have now specified the i-level dynamics. In this section we consider the p-level (p for population) and the feedback to the i-level via the variables $F_\pm$ and $\varLambda _\pm$.

#### Bookkeeping

In a deterministic description of a large population

12 $$\begin{aligned} P_\ell (t)=\int _0^\infty \mu e^{-\mu a}p_\ell (t-a,a)da=\int _{-\infty }^t\mu e^{-\mu (t-\alpha )}p_\ell (\alpha ,t-\alpha )d\alpha , \end{aligned}$$

is the fraction of the population that is in state $\ell$ at time $t$. In Sect. 3.3 we rewrite these identities as differential equations.

#### Feedback

It remains to provide the feedback relations that express the individual level input variables $F_\pm (t)$ and $\varLambda _\pm (t)$ in terms of output variables at the population level. Directly from the interpretation it follows that we should take

13 $$\begin{aligned} F_\pm (t)=\frac{1}{n}\sum _{k_+=0}^{n}\sum _{k_-=0}^{n-k_+}(n-k_{-}-k_+)P_{(\pm ,k_-,k_+)}(t). \end{aligned}$$

The only unknown terms left are the mean field at distance one rates $\varLambda _\pm (t)$. In the remainder of this section we define these rates and explain why our description is not exact.

Consider a transition of an individual $u$, with $u$ in state $(\pm ,k_-,k_+)\rightarrow (\pm ,k_{-}-1,k_++1)$. This transition occurs when a susceptible partner $v$ of the focus individual $u$ in state $(\pm ,k_-,k_+)$ gets infected. The rate at which $v$ gets infected depends on the number of infectious partners $v$ has. However, we only know that $v$ is a susceptible partner of $u$.

Note that we can not distinguish between two susceptible partners $v_1$ and $v_2$ of an individual $u$ and that the states of $v_1$ and $v_2$ are correlated in the same way with the state of $u$. In particular, the probability that $v_1$ is in state $(-,m_-,m_+)$ is equal to the probability that $v_2$ is in that state. Therefore, we are interested in probabilities $\lambda (m_+|(\pm ,k_-,k_+))$, where $\lambda (m_+|(\pm ,k_-,k_+))$ denotes the conditional probability that a susceptible partner of an individual in state $(\pm ,k_-,k_+)$ has itself $m_+$ infectious partners. The force of infection on a susceptible individual with $m_+$ partners is $\beta m_+$. Therefore, by averaging over all possibilities, we obtain the following rates for the corresponding transitions:

$$\begin{aligned} (-,k_-,k_+)&\rightarrow (-,k_{-}-1,k_++1)\quad \text { with rate }k_-\sum _{m_+=0}^{n-1}\beta m_+\lambda (m_+|(-,k_-,k_+)),\\ (+,k_-,k_+)&\rightarrow (+,k_{-}-1,k_++1)\quad \text { with rate }k_-\sum _{m_+=1}^{n}\beta m_+\lambda (m_+|(+,k_-,k_+)). \end{aligned}$$

We now make the simplifying assumption that the probability that a susceptible partner of $u$ has $m_+$ infectious partners does not depend on the exact state of $u$ but only on $u$ being susceptible or infectious. More precisely, we assume that we can approximate $\lambda (m_+|(\pm ,k_-,k_+))$ by

$$\begin{aligned} \lambda _\pm (m_+), \end{aligned}$$

where $\lambda _-(m_+)$ is the conditional probability that $v$ has $m_+$ infectious partners, given that $v$ is susceptible and $v$ is a partner of susceptible individual $u$ and $\lambda _+(m_+)$ is that same conditional probability when $v$ is a partner of infectious individual $u$. In fact, as we explain in Appendix B, the probabilities $\lambda _\pm (m_+)$ are really an approximation of $\lambda (m_+|(\pm ,k_-,k_+))$ as these probabilities ignore correlations of $u$ and $v$, i.e. between the states of two individuals that are in a partnership. Note that for certain static networks one can actually justify the mean field at distance one assumption for ${ SI}$ and ${ SIR}$ infection (but presumably not for ${ SIS}$), see (Decreusefond et al. 2012; Barbour and Reinert 2013).

Assuming a two-type version of (8) we define

14 $$\begin{aligned} \lambda _-(m_+)=\frac{\sum _{m_-=1}^{n-m_+}m_-P_{(-,m_-,m_+)}(t)}{\sum _{l_+=0}^{n-1}\sum _{l_-=1}^{n-l_+}l_-P_{(-,l_-,l_+)}(t)}, \end{aligned}$$

with the convention that $\lambda _-(m_+)=0$ if the denominator equals zero, and

15 $$\begin{aligned} \lambda _+(m_+)=\frac{\sum _{m_-=0}^{n-m_+}m_+P_{(-,m_-,m_+)}(t)}{\sum _{l_+=1}^n\sum _{l_-=0}^{n-l_+}l_+P_{(-,l_-,l_+)}(t)}, \end{aligned}$$

with the convention that $\lambda _+(1)=1$ and $\lambda _+(m_+)=0$ for $m_+>1$, if the denominator equals zero. The explanation of (14) and (15) is as follows. In both cases, we consider the probability that the state of $v$ is $(-,m_-,m_+)$, given that $v$ is susceptible and $v$ has a partner $u$. In the case of (14), $u$ is susceptible, so, if we also take into account that $u$ is one of the $m_-$ susceptible partners of $v$, the probability that $v$ is in state $(-,m_-,m_+)$ is

$$\begin{aligned} \frac{m_-P_{(-,m_-,m_+)}(t)}{\sum _{l_+=0}^{n-1}\sum _{l_-=1}^{n-l_+}l_-P_{(-,l_-,l_+)}(t)} \end{aligned}$$

cf. Lemma 2. Similarly, in the case of (15), we ‘arrive’ at $v$ via its link to the *infectious* $u$, so then the probability that $v$ is in state $(-,m_-,m_+)$ is

$$\begin{aligned} \frac{m_+P_{(-,m_-,m_+)}(t)}{\sum _{l_+=0}^{n-1}\sum _{l_-=1}^{n-l_+}l_+P_{(-,l_-,l_+)}(t)}. \end{aligned}$$

In both cases, the denominator serves to normalize.

The mean field at distance one terms $\varLambda _\pm$ in (9) are now specified by

16 $$\begin{aligned} \varLambda _-(t)=\sum _{m_+=1}^{n-1}m_+\lambda _-(m_+) \end{aligned}$$

with $\lambda _-(m_+)$ given by (14), and

17 $$\begin{aligned} \varLambda _+(t)=\sum _{m_+=1}^n m_+\lambda _+(m_+)=1+\sum _{m_+=2}^n(m_+-1)\lambda _+(m_+), \end{aligned}$$

with $\lambda _+(m_+)$ given by (15). (For mean field at distance one terms also see Lindquist et al. 2011.)

Note that, from an individual-based perspective, (16) and (17) are the only formulas consistent with our assumption that $u$’s susceptible partners are subject to a force of infection $\beta \varLambda _\pm$ depending only on $t$ and $u$’s infection status $\pm$ (and not on the number of susceptible and infectious partners of $u$ cf. Appendix B). Hence our choice of the term ‘mean field at distance one’ for the latter assumption.

### The p-level differential equations

In a deterministic description of a large population, $P_{(\pm ,k_-,k_+)}$ denotes the fraction of the population in state $(\pm ,k_-,k_+)$ We take as the convention that the $P_{(\pm ,k_-,k_+)}$ should be interpreted as zero when $k_-+k_+>n$, $k_-<0$, or $k_+<0$. By differentiation of (12) and using (10)–(11) for $p$, we obtain the following set of $(n+1)(n+2)$ differential equations:

$$\begin{aligned} \frac{dP_{(-,0,0)}}{dt}&=\mu -(\rho \bar{F} n+\mu )P_{(-,0,0)}+(\sigma +\mu )(P_{(-,1,0)}+P_{(-,0,1)})\\ \frac{dP_{(-,k_-,k_+)}}{dt}&=-\left( \rho \bar{F}(n-k_{-}-k_+)+(\sigma +\mu )(k_-+k_+)\right. \\&\quad \left. +\beta k_++\beta \varLambda _-k_-+\mu \right) P_{(-,k_-,k_+)}\\&+\rho F_-(n-k_{-}-k_++1)P_{(-,k_{-}-1,k_+)}\\&+\rho F_+(n-k_{-}-k_++1)P_{(-,k_-,k_+-1)}\\&+(\sigma +\mu )\left( (k_-+1)P_{(-,k_-+1,k_+)}+(k_++1)P_{(-,k_-,k_++1)}\right) \\&+\beta \varLambda _-(k_-+1)P_{(-,k_-+1,k_+-1)}\\ \frac{dP_{(+,k_-,k_+)}}{dt}&{=}{-}\left( \rho \bar{F} (n-k_{-}-k_+)+(\sigma +\mu )(k_-+k_+){+}\beta \varLambda _+k_-+\mu \right) P_{(+,k_-,k_+)}\\&+\rho F_-(n-k_{-}-k_++1)P_{(+,k_{-}-1,k_+)}\\&\quad +\rho F_+(n-k_{-}-k_++1)P_{(+,k_-,k_+-1)}\\&+(\sigma +\mu )\left( (k_-+1)P_{(+,k_-+1,k_+)}+(k_++1)P_{(+,k_-,k_++1)}\right) \\&+\beta \varLambda _+(k_-+1)P_{(+,k_-+1,k_+-1)}+\beta k_+P_{(-,k_-,k_+)}. \end{aligned}$$

Choose the same ordering of the $\ell$’s as before with the i-states in Sect. 3.2 and let $P$ denote the corresponding vector of the variables $P_\ell$. In matrix notation, we have

18 $$\begin{aligned} \frac{dP}{dt}=\mu \mathbf {1}_{{(-,0,0)}}+B\left( F_\pm ,\varLambda _\pm \right) P-\mu P, \end{aligned}$$

where $\mathbf {1}_{{(-,0,0)}}$ is the indicator function of ${(-,0,0)}$, and $B$ is the matrix corresponding to the rates of the state transitions described in Sects. 3.1.1 and 3.1.2.

#### Consistency relations

The $P_\ell$ are related to each other by:

19 $$\begin{aligned} \sum _{k_+=0}^n\sum _{k_-=0}^{n-k_+}k_+P_{(-,k_-,k_+)}(t)=\sum _{k_+=0}^n\sum _{k_-=0}^{n-k_+}k_-P_{(+,k_-,k_+)}(t), \end{aligned}$$

This is evident from the interpretation, since both terms denote the number of ${ SI}$ partnerships, i.e. the number of partnerships involving an infectious and a susceptible individual. The proof of (19) starts by differentiating both left- and right hand side with respect to $t$ and continues by inserting components of (18); this is worked out for a similar situation in (Lindquist et al. 2011, Appendix B).

We have assumed that the infectious disease has no influence on the partnership formation and separation or on the probability per unit of time of dying. Therefore, the disease-free partnership network is embedded in (18) and the fraction of individuals in the population in state $k$ at time $t$ is equal to

20 $$\begin{aligned} P_k(t)=\sum _{k_-+k_+=k}\left( P_{(-,k_-,k_+)}(t)+P_{(+,k_-,k_+)}(t)\right) . \end{aligned}$$

Furthermore, the dynamics of partnerships in the population are governed by the sum of the fraction of free susceptible and the fraction of free infectious binding sites, which is equal to the total fraction of free binding sites, i.e. $F_-(t)+F_+(t)=F(t)$. As a consequence, the set characterized by

21 $$\begin{aligned} F_-(t)+F_+(t)=\bar{F} \end{aligned}$$

is invariant and attracting. Therefore, also in the network with infection superimposed, we consider $F(t)$ constant and equal to $\bar{F}$ (see Lemma 1). Likewise, we can consider the left hand side of (20) as constant in time and given by (6).

## Linearisation and the map $L$

In this section we linearise system (18) around the disease-free equilibrium. Next we show that we can reduce the dimension of the linearised system and consider only the variables $P_{(-,k_-,1)}$ and $P_{(+,k_-,k_+)}$. In Sect. 6 we will use this reduced linearised system to prove that the basic reproduction number $R_0$, that we characterize in Sect. 5, indeed provides a threshold value of 1 for the disease free steady state of system (18) to become unstable. To this end we define a map $L$ in Sect. 4.2, which allows us to relate, in the linearisation, population-level fractions of individuals (that we consider in the present section) to fractions of binding sites (that we consider in Sect. 5).

### Linearisation

Note that the disease-free equilibrium is given by

$$\begin{aligned} P_{(-,k,0)}(t)=P_k, \end{aligned}$$

$0\le k\le n$, and $P_\ell (t)=0$ for all triplets $\ell$ not of the form $(-,k,0)$.

Next, note that we can use relationship (21) in order to replace $F_-$ by $\bar{F}-F_+$ (note that this last expression does not involve any variable of the form $P_{(-,k,0)}$). Next, we can reduce the dimension of the system by $n+1$ by eliminating the $P_{(-,k,0)}$, $k=0,\ldots ,n$, from the system using relation (20).

Consider the differential equations for $P_{(-,k_-,1)}$, $0\le k_-\le n-1$, explicitly given by

$$\begin{aligned} \frac{dP_{(-,k_-,1)}}{dt}&={-}\left( \rho \bar{F}(n-k_{-}{-}1)+(\sigma +\mu )(k_-+1)+\beta +\beta \varLambda _-k_-+\mu \right) P_{(-,k_{-},1)}\\&+\rho (\bar{F}-F_+)(n-k_-)P_{(-,k_{-}-1,1)}+\rho F_+(n-k_-)P_{(-,k_-,0)}\\&+(\sigma +\mu )\left( (k_-+1)P_{(-,k_-+1,1)}+2P_{(-,k_-,2)}\right) \\&+\beta \varLambda _-(k_-+1)P_{(-,k_-+1,0)} \end{aligned}$$

(as one can verify by writing out the relevant part of (18)).

Then the only nonlinear terms are those that involve $F_+$ or $\varLambda _\pm$ as a factor. In these differential equations we find, among the nonlinear terms,

22 $$\begin{aligned} \rho F_+(n-k_-)P_{(-,k_-,0)} \end{aligned}$$

and

23 $$\begin{aligned} \beta \varLambda _-(k_-+1)P_{(-,k_-+1,0)}. \end{aligned}$$

Trusting that it does not lead to confusion we will denote the variables in the linearisation of (18) by the same symbols as the variables in the nonlinear system.

Linearisation of (22) yields

$$\begin{aligned} \rho F_+(n-k_-)P_{k_-} \end{aligned}$$

where $P_{k_-}$ is the fraction of the population in state $k_-$ in the disease-free network and $F_+$ is defined as in (13), only now for the variables of the linearised system. For (23), similarly replace $P_{(-,k_-+1,0)}$ by $P_{k_-+1}$ but next use the identity

$$\begin{aligned} (k_-+1)P_{k_-+1}=Q_{k_-+1}\sum _{m}mP_m \end{aligned}$$

(cf. (8)). In the definition (16) of $\varLambda _-$ we take linearisation into account by adapting the denominator of the expression for $\lambda _-$ in (14). More precisely, we replace that denominator by

$$\begin{aligned} \sum _mmP_m. \end{aligned}$$

Note that this cancels the identical factor in the numerator. The upshot is that this sum leads to the linearisation of (23) being equal to

24 $$\begin{aligned} \beta Q_{k_-+1}\sum _{j_+=0}^{n-1}\sum _{j_-=1}^{n-j_+}j_+j_-P_{(-,j_-,j_+)}. \end{aligned}$$

In all other nonlinear terms, whenever $F_+$ or $\varLambda _\pm$ multiplies $P_\ell$ and $P_\ell$ is zero in the disease free steady state, simply put $F_+$ respectively $\varLambda _\pm$ equal to their values in the disease-free equilibrium, i.e.

$$\begin{aligned} F_+&=0\\ \varLambda _-&=0\\ \varLambda _+&=1, \end{aligned}$$

to obtain the corresponding term for the linearised system.

Thus we deduce that the linearised system is given by25


#### *Remark 1*

In Lemma 3 below we will show that we can simplify expression (24) to

$$\begin{aligned} \beta Q_{k_-+1}\sum _{j_-=0}^{n-1}j_-P_{(-,j_-,1)}. \end{aligned}$$

Intuitively, one would expect that, in the linearisation, for $k_+\ge 2$, $P_{(-,k_-k_+)}(t)=0$ for all $t$ if $P_{(-,k_-k_+)}(0)=0$. Indeed, in the beginning of an epidemic very few individuals in the population are infectious. It is already very unlikely for a susceptible individual to have an infectious partner, so the probability that a susceptible individual has more than one infectious partner should be negligible. That this is indeed the case, is established in the following lemma.

#### **Lemma 3**

In the linearised system (25), if $P_{(-,k_-k_+)}(0)=0$, then

$$\begin{aligned} P_{(-,k_-,k_+)}(t)\equiv 0, \end{aligned}$$

for $$k_+\ge 2$$.

#### *Proof*

We prove the lemma in four steps

**Fig. 1:**
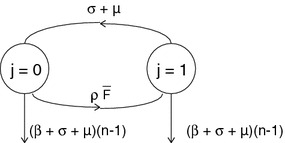
Diagram that shows that, if $P_{(-,j,n-1)}(0)=0$, then $P_{(-,j,n-1)}\equiv 0$, $j=0,1$

**Fig. 2:**
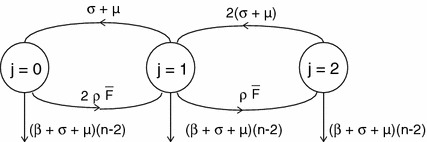
Diagram that shows that, if $P_{(-,j,n-2)}(0)=0$, then $P_{(-,j,n-2)}\equiv 0$, $j=0,1,2$

Step 1.: Observe first that the differential equations for $P_{(-,k_-,k_+)}$, $k_+\ge 2$, form a closed system, i.e. they do not depend on the remaining variables (see (25)).
Step 2.: Observe that this closed system has a certain hierarchical structure, viz. the subsystem for the variables
  $$\begin{aligned} P_{(-,j,n-k)}, \end{aligned}$$
  $0\le j\le k$, depends on the variables of the subsystems with a lower value of $k$, but not on the variables of any subsystem with a higher value of $k$ (the reason is that both $F_+$ and $\varLambda _-$ were put equal to zero to derive the equations that we consider; recall that we focus on $n-k\ge 2$).
Step 3.: For $k=0$ we have
  $$\begin{aligned} \frac{dP_{(-,0,n)}}{dt}=-\left( (\sigma +\mu )n+\mu +\beta n\right) P_{(-,0,n)} \end{aligned}$$
  so, if $P_{(-,0,n)}(0)=0$, then $P_{(-,0,n)}\equiv 0$.
Step 4.: Consider $k=1$. The diagram in Fig. 1 shows at once that the zero state is globally stable, i.e. if $P_{(-,j,n-1)}(0)=0$, then $P_{(-,j,n-1)}\equiv 0$, $j=0,1$. For $k=2$, we have the diagram in Fig. 2, which shows that if $P_{(-,j,n-2)}(0)=0$, then $P_{(-,j,n-2)}\equiv 0$, $j=0,1,2$.

By continuing in this way we establish that for all $k$ with $0\le k\le n-2$, if $P_{(-,j,n-k)}(0)=0$, then $P_{(-,j,n-k)}\equiv 0$, $j=0,1,\ldots ,k$. $\square$

It follows that we are left to deal with the stability of the following linear system:26Recall definition (13) of $F_+$. In the reduced linearised system (26) we are left with variables $P_{(-,k_-,1)}$ and $P_{(+,k_-,k_+)}$, $k_-,k_+\ge 0$, $0\le k_-+k_+\le n$. Therefore, (26) is a closed system. Furthermore, note that the dimension of the system is $n+\frac{1}{2}(n+1)(n+2)=\frac{1}{2}(n^2+5n+2)$ (where the contribution $n$ comes from the $P_{(-,k_-,1)}$ and the $\frac{1}{2}(n+1)(n+2)$ from the $P_{(+,k_-,k_+)}$).

### The map $L$

Order the $P_\ell$ in some appropriate way, and denote the corresponding vector by $P$. We define a linear map $L$ from ${{\mathrm{\mathbb {R}}}}^{\frac{1}{2}(n^2+5n+2)}$ to ${{\mathrm{\mathbb {R}}}}^{n+2}$ as follows:

27 $$\begin{aligned} L(P)=\begin{pmatrix}\sum _{k_+=0}^n\sum _{k_-=0}^{n-k_+}(n-k_--k_+)P_{(+,k_-,k_+)}\\ \left( P_{(-,j-1,1)}\right) _{j=1}^n\\ \sum _{k_+=0}^n\sum _{k_-=0}^{n-k_+}k_+P_{(+,k_-,k_+)} \end{pmatrix}. \end{aligned}$$

Note that $L$ maps the positive $P$-cone to the positive cone in ${{\mathrm{\mathbb {R}}}}^{n+2}$. In fact, if $P$ is in the interior of the positive cone, i.e. all vector elements are strictly positive, then

$$\begin{aligned} \sum _{k_+=0}^n\sum _{k_-=0}^{n-k_+}(n-k_--k_+)P_{(+,k_-,k_+)}>0, \end{aligned}$$

since $n-k_--k_+\ge 0$ for all $k_-+k_+< n$, and $P_{(+,k_-,k_+)}>0$ for all $k_-$ and $k_+$,

$$\begin{aligned} P_{(-,j,1)}>0, \end{aligned}$$

and

$$\begin{aligned} \sum _{k_+=0}^n\sum _{k_-=0}^{n-k_+}k_+P_{(+,k_-,k_+)}>0, \end{aligned}$$

since $P_{(+,k_-,k_+)}>0$ for all $k_-$ and we sum over $k_+=0,1,2,\ldots ,n$. In particular it follows that if $L(P)=0$, then $P=0$. We shall use this linear operator $L$ in Sect. 6.

## Dynamics of the binding sites of an infectious individual: characterization of $R_0$

By exploiting that an individual can be considered as a collection of $n$ binding sites that behave independently from one another as far as separation or acquiring a new partner is concerned and by using our mean field at distance one assumption, we are able to characterize $R_0$ in terms of binding sites. In this section we only use the interpretation of the model and we do not use the system (18) or its reduced linearisation (26). We characterize $R_0$ as the dominant eigenvalue of a next-generation matrix (NGM) that we construct using the interpretation of the model.

The entries in the NGM can be viewed as expected offspring values for a multi-type branching process (Jagers 1975; Haccou et al. 2005), with the two matrix-indices specifying the type at birth of, respectively, offspring and parent. Several slightly different branching processes may yield the same NGM and for the deterministic theory (which is what we deal with here) there is no need to choose one of these as ‘the’ underlying process. A branching process corresponding to the NGM is subcritical when $R_0<1$ and supercritical when $R_0>1$. But does such a branching process indeed correspond to the linearisation of (18) in the disease free steady state? Especially for $n>1$ this is a nontrivial question. In Sect. 6 we will therefore prove that $R_0$, as computed from the NGM, is indeed a threshold parameter with threshold value one for (18).

First, in Sect. 5.1, we consider the case $n=1$. In Sect. 5.2 we generalize the transition and transmission scheme to $n>1$, and in Sect. 5.3 we characterize $R_0$ on the level of binding sites. We conclude this section by showing in Sect. 5.4 that $R_0$ also has an interpretation in terms of individuals. The explicit expression for $R_0$ and the remainder of its derivation is left for Appendix C.

Consider the usual setting for determining $R_0$, i.e. suppose that we have a population in which only a few individuals are infectious and all others are susceptible. We are interested in the expected number of secondary cases caused by one ‘typical’ infectious case.

### The case $n=1$

First, consider a population of individuals with partnership capacity $n=1$. Then each individual has exactly one binding site. If we now consider an infectious individual, then its binding site can be in one of three states:

- $$A$$—free
- $B$—occupied by a susceptible partner
- $C$—occupied by an infectious partner

**Fig. 3:**
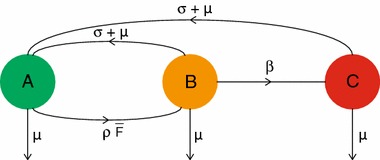
Flow chart describing the possible transitions between states $A$, $B$ and $C$ and their corresponding rates. Note that, in the beginning of the epidemic, only a few individuals in the population are infectious. Therefore the probability that an infectious individual acquires an infectious partner is zero. This is represented in the flowchart where there is no direct *arrow* from $A$ to $C$

Please note that we recycle symbols: the $A$ here has nothing to do with the matrix $A$ of Sect. 2 and the $B$ here has nothing to do with the matrix $B$ in (10) of Sect. 3. In Fig. 3 the possible state transitions and corresponding rates for an infectious individual are given. Note that it is highly unlikely that an infectious individual acquires an infectious partner in the beginning of an epidemic, and therefore there is no transition from $A$ to $C$.

We can characterize $R_0$ by constructing an NGM $K_1$ that involves a transmission part $T_1$ and a transition part $\varSigma _1$.

Recall that we use the convention that, for a transition matrix $M=(m_{ij})$, $m_{ij}$ denotes the probability per unit of time at which a transition from $j$ to i occurs (instead of the transition from i to $j$, as it is common in the stochastic community).

The matrices $T_1$ and $\varSigma _1$ are obtained as follows. Consider an infectious individual, and order the states as $A$, $B$, $C$. Then the transitions of the individual’s binding site are described by the following matrix $\varSigma _1$ (see Fig. 3 for its graphical representation):

28 $$\begin{aligned} \varSigma _1=\begin{pmatrix}-(\rho \bar{F}+\mu ) &{} \sigma +\mu &{}\sigma +\mu \\ \rho \bar{F} &{} -(\beta +\sigma +2\mu )&{} 0\\ 0&{}\beta &{}-(\sigma +2\mu )\end{pmatrix}. \end{aligned}$$

Here $(\varSigma _1)_{xy}$ is the rate at which a transition from a binding site in state $y$ to state $x$ occurs, $x,y\in \{A,B,C\}$, $x\ne y$, and for the diagonal elements we have $(\varSigma _1)_{xx}=-(\mu +\sum _{y\ne x}(\varSigma _1)_{yx})$.

Consider an infectious individual $u$ with its binding site in state $B$. If $u$ infects its susceptible partner $v$, then the binding site of $u$ transits from $B$ to $C$. This transition is represented by $(\varSigma _1)_{CB}=\beta >0$. In addition to this transition, an additional $C$ binding site is created. Indeed, $v$ is now also an infectious individual who has a binding site occupied by an infectious partner (namely $u$). This shows that one transition from $B$ to $C$ always creates one additional $C$ binding site in the population. Accordingly we define the transmission matrix $T_1$:

29 $$\begin{aligned} T_1=\beta \begin{pmatrix}0&{}\quad 0&{}\quad 0\\ 0&{}\quad 0&{}\quad 0\\ 0&{}\quad 1&{}\quad 0\end{pmatrix}. \end{aligned}$$

Using $T_1$ and $\varSigma _1$ we can construct the NGM $K_1$:

$$\begin{aligned} K_1=T_1(-\varSigma _1)^{-1}. \end{aligned}$$

The basic reproduction number $R_0$ is defined as the dominant eigenvalue of $K_1$ (Diekmann et al. 2013, Section 7.2).

In the present case we can, quite easily, give an explicit expression for $R_0$. Note that $T_1$ has one-dimensional range spanned by the vector $(0,0,1)'$. Therefore $(0,0,1)'$ is the eigenvector corresponding to the dominant eigenvalue $R_0$. We find $K_1$ applied to this vector by first constructing $(-\varSigma _1)^{-1}$ applied to this vector. This can be done by either treating it as a linear algebra problem or we can use the interpretation for it: $(-(\varSigma _1)^{-1}(0,0,1)')_x$ is the mean time spent in state $x$ when starting in state $C$, $x=A$, $B$, $C$ (in fact we only use $x=B$). We find that

$$\begin{aligned} (-\varSigma _1)^{-1}\begin{pmatrix}0\\ 0\\ 1\end{pmatrix}=\begin{pmatrix}\frac{\sigma +\mu }{\mu (\rho \bar{F}+\sigma +2\mu )}\\ \frac{\rho \bar{F}(\sigma +\mu )}{\mu (\beta +\sigma +2\mu )(\rho \bar{F}+\sigma +2\mu )}\\ \frac{\beta (\rho \bar{F}+\mu )+\mu (\rho \bar{F}+\sigma +2\mu )}{\mu (\beta +\sigma +2\mu )(\rho \bar{F}+\sigma +2\mu )}\end{pmatrix}, \end{aligned}$$

and subsequently,

$$\begin{aligned} K_1\begin{pmatrix}0\\ 0\\ 1\end{pmatrix}=\frac{\beta \rho \bar{F}(\sigma +\mu )}{\mu (\beta +\sigma +2\mu )(\rho \bar{F}+\sigma +2\mu )}\begin{pmatrix}0\\ 0\\ 1\end{pmatrix}, \end{aligned}$$

from which we conclude that

30 $$\begin{aligned} R_0=\frac{\beta \rho \bar{F}(\sigma +\mu )}{\mu (\beta +\sigma +2\mu )(\rho \bar{F}+\sigma +2\mu )}. \end{aligned}$$

Alternatively, we can characterize $R_0$ by first step analysis; see Appendix A for the details or Diekmann et al. (2013, Section 7.8) or Miller et al. (2012, formula (3.1.9)) or Inaba (1997, Section 4.1). However, this does not have such a nice generalization to $n>1$ as the $ABC$ scheme does.

### Generalization of the transition and transmission matrix: $n>1$

Now consider the case $n>1$. In this case, an individual is a collection of $n$ binding sites. These binding sites may be free, occupied by a susceptible or occupied by an infectious individual, i.e. in states $A$, $B$, or $C$, respectively. An infectious individual can infect a susceptible individual in the population if it has a binding site that is occupied by a susceptible individual. In that case, that binding site becomes occupied by an infectious individual. Similar to the $n=1$ situation we observe that if a binding site makes a transition from ‘occupied by a susceptible individual’ to $C$, it creates a new infectious individual in the population. However, we need to know in which states the $n$ binding sites of this new infectious individual are. Obviously, one new infectious binding site is in state $C$, viz. the binding site still occupied by its epidemiological parent. In order to know the states of the other $n-1$ binding sites, we need to know the number of (susceptible) partners of this individual at epidemiological birth.

Naively, motivated by Lemma 2, one would think (as we did at first) that the number of partners of a newly infected individual is $k$ (i.e. 1 binding site in state $C$,   $k-1$ binding sites in state $B$ and $n-k$ binding sites in state $A$) with probability $Q_k$. The computation of the corresponding $R_0$ is rather straightforward (using the method explained in Appendix A for $n=1$). However, one can check numerically that the stability switch of the disease free steady state of (18) does *not* coincide with $R_0=1$ when $R_0$ is defined in this manner. We conclude that the premise is wrong. In retrospect this makes sense. First of all, we know that $q$ differs from $Q$, where $q$ and $Q$ are defined by (7) and (8), respectively. In our model description we keep track of the number of partners of an individual. We use mean field at distance one for the partners of partners of this individual (and this shows up in the $\varLambda _\pm$ in the transmission events). So we need to do the same when characterizing $R_0$ and also take into account the partners of susceptible partners. Therefore, we need to extend the information that is tracked in the scheme.

We generalize the $ABC$ scheme of Sect. 5.1 as follows. Consider an infectious binding site. Then this binding site can be in one of $n+2$ states:

- $$A$$—free
- $B_j$—occupied by a susceptible partner that has $j$ partners in total, $j=1,\ldots , n$
- $C$—occupied by an infectious partner.

**Fig. 4:**
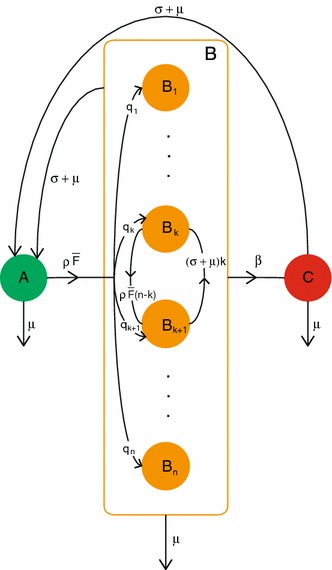
Flow chart describing the possible transitions between states $A$, $B_j$, $j=1,\ldots ,n$, and $C$ and their corresponding rates

Let ${{\varvec{B}}}$ denote the collection of all states $B_j$. We denote the transition matrix of the states $A$, $B_j$, $j=1,\ldots ,n$, and $C$ by $\varSigma$ (see Fig. 4 for the corresponding flowchart), where

31 $$\begin{aligned} \varSigma =\begin{pmatrix}-(\rho \bar{F}+\mu )&{}\quad \varvec{\sigma } +\varvec{\mu }&{}\quad \sigma +\mu \\ \rho \bar{F} {\varvec{q}}&{}\quad \varSigma _{{\varvec{B}}} &{}\quad \varvec{0}\\ 0&{}\quad \varvec{\beta }&{}\quad -(\sigma +2\mu )\end{pmatrix}, \end{aligned}$$

where $\varvec{0}$ denotes the $n$ dimensional zero vector, $\varvec{\sigma } +\varvec{\mu }$ and $\varvec{\beta }$ both denote an $n$-dimensional row vector, namely

$$\begin{aligned} \varvec{\sigma } +\varvec{\mu }&=(\sigma +\mu )\begin{pmatrix}1&1&\cdots&1\end{pmatrix},\\ \varvec{\beta }&=\beta \begin{pmatrix}1&1&\cdots&1\end{pmatrix}. \end{aligned}$$

The vector ${\varvec{q}}$ is the probability vector with elements $q_k$ given by (7), and $\varSigma _{{\varvec{B}}}$ is an $n\times n$ matrix describing the transitions between the states $B_1$, $\ldots$, $B_n$ and out of ${\varvec{B}}$; see Fig. 4 for the corresponding flowchart.

Let’s describe $\varSigma _{{\varvec{B}}}$ more carefully. The matrix $\varSigma _{{\varvec{B}}}$ describes the transitions between the $B_j$ and out of ${\varvec{B}}$. Thus $\varSigma _{{\varvec{B}}}$ is an $n\times n$ tridiagonal matrix with negative diagonal entries and positive off-diagonal entries. More specifically,

$$\begin{aligned} (\varSigma _{{\varvec{B}}})_{j-1,j}&= \ (\sigma +\mu )(j-1),\\ (\varSigma _{{\varvec{B}}})_{j,j}&= \ -(\beta +\rho \bar{F}(n-j)+(\sigma +\mu )j+\mu ),\\ (\varSigma _{{\varvec{B}}})_{j+1,j}&= \ \rho \bar{F}(n-j). \end{aligned}$$

Indeed, a susceptible individual with 1 infectious and $j-1$ susceptible partners loses one of these susceptible partners at rate $(\sigma +\mu )(j-1)$, acquires a new susceptible partner at rate $\rho \bar{F}(n-j)$, and, since it can also become infectious, lose its infectious partner, or die (these last three mark transitions out of ${\varvec{B}}$), the rate out of $B_j$ is $(\varSigma _{{\varvec{B}}})_{j,j}=\ -(\beta +\rho \bar{F}(n-j)+(\sigma +\mu )j+\mu )$.

The other elements of $\varSigma$ have the following interpretation. Note that, in the beginning of an epidemic, a binding site in state $A$ acquires a susceptible partner at rate $\rho \bar{F}$. The probability that, just after the moment of acquisition, this susceptible partner has in total $j$ partners is $q_j$ in accordance with (7). Therefore, the rate at which a binding site in state $A$ transits to state $B_j$ is $(\varSigma )_{B_j,A}=\rho \bar{F} q_j$. In a similar way one can use the interpretation (and the flowchart in Fig. 4) to find the other entries for the matrix $\varSigma$.

**Fig. 5:**
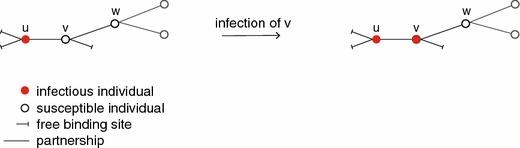
Illustration of the construction of $T$ for $n=3$. Suppose we start with an individual with two binding sites in $A$ and one binding site in $B_2$. Then $u$ has one susceptible partner $v$. If $u$ infects $v$, then $v$ will have one binding site in $C$, one binding site in $A$, and one binding site will be occupied by a susceptible partner $w$. In the example, $w$ has three partners in total and therefore the binding site of $v$ would be in state $B_3$. However, information about the partners of $w$ is not incorporated in our model description and therefore we assume that $w$ has three partners with probability $Q_3$

Finally, we need to construct the transmission matrix $T$. A transmission corresponds to a transition $B_j\rightarrow C$, i.e. if an infectious individual $u$ with a binding site in $B_j$ infects its partner $v$. This is included in the matrix $\varSigma$ since $(\varSigma )_{C,B_j}=\beta >0$. The transmission matrix $T$ includes the binding sites of the newly infected partner $v$. Concerning the binding sites of $v$, since it is now infectious, we observe that it has one binding site in $C$, $n-j$ binding sites in $A$ and $j-1$ binding sites will be occupied by susceptible individuals, i.e. $j-1$ binding sites will be in the set ${\varvec{B}}$ (see Fig. 5 for an illustration where $u$ has a binding site in $B_2$ that changes state to $C$ and $v$ is the newly infected individual with one binding site in $C$, one binding site in $A$ and one binding site occupied by a susceptible individual). All that is left to specify are the states of the $j-1$ binding sites in ${\varvec{B}}$, i.e. we need to know how many partners these susceptible partners of $v$ have (in Fig. 5: how many partners does $w$ have).

The probability that a partner $w$ of $v$ has $k$ partners depends on the state of $v$, where $v$ is in state $(+,j-1,1)$ immediately after infection by $u$. However, as another manifestation of the mean field at distance one assumption, we approximate this probability by only taking into account that the susceptible individual $w$ has at least one partner $v$. Therefore, we assume that $w$ has $k$ partners with probability $Q_k$ (cf. Lemma 2). In other words, we assume that a binding site of $v$ occupied by a susceptible partner, i.e. a binding site in the set ${\varvec{B}}$, is in state $B_k$ with probability $Q_k$.

Accordingly, we define the transmission matrix $T$ as follows:

32 $$\begin{aligned} T=\beta \left( \begin{array}{ccccc}0&\phi _1&\cdots&\phi _n&0\end{array}\right) , \end{aligned}$$

where $\phi _j$ is the $n+2$ vector

33 $$\begin{aligned} \phi _j=\left( \begin{array}{c}n-j \\ (j-1)\varvec{Q} \\ 1\end{array}\right) =(n-j)\psi _A+(j-1)\psi _{{\varvec{B}}}+\psi _C, \end{aligned}$$

$$j=1,\ldots ,n$$, where

34 $$\begin{aligned} \psi _A=\left( \begin{array}{c}1 \\ \varvec{0} \\ 0\end{array}\right) , \quad \psi _{{\varvec{B}}}=\left( \begin{array}{c}0\\ \varvec{Q}\\ 0\end{array}\right) , \quad \psi _C=\left( \begin{array}{c}0\\ \varvec{0}\\ 1\end{array}\right) , \end{aligned}$$

and ${\varvec{Q}}$ is the probability vector with components $Q_k$ given by (8). Note that the $\phi _j$ are a linear combination of the $\psi _x$, $x\in \{A,{{\varvec{B}}},C\}$. We conclude that the range of $T$ is spanned by $\psi _A$, $\psi _{{\varvec{B}}}$, $\psi _C$.

In Sect. 5.4 we shall show that we can identify the $\phi _j$ with an individual in state $(+,j-1,1)$, which allows us to interpret $R_0$ in terms of individuals. But first, in Sect. 5.3, we focus on the interpretation in terms of binding sites.

### $R_0$ in terms of binding sites

Now that we have defined the transition matrix $\varSigma$ and the transmission matrix $T$, we are ready to define the basic reproduction ratio $R_0$ for $n>1$ as the dominant eigenvalue of the matrix

$$\begin{aligned} T(-\varSigma )^{-1}. \end{aligned}$$

In order to underpin this, consider variables $X_A$, $X_{B_j}$, and $X_C$, where $X_A$, $X_{B_j}$, and $X_C$ are the fractions of the total binding-site population in states $A$, $B_j$, and $C$, respectively. Then, based on the interpretation, $X_A$, $X_{B_j}$, and $X_C$ should satisfy the following system of differential equations:

35 $$\begin{aligned} \frac{d}{dt}\begin{pmatrix}X_A\\ X_{B_1}\\ \vdots \\ X_{B_n}\\ X_C\end{pmatrix}= (T+\varSigma )\begin{pmatrix}X_A\\ X_{B_1}\\ \vdots \\ X_{B_n}\\ X_C\end{pmatrix}\!. \end{aligned}$$

It follows that the zero state $(X_A,X_{B_1},\ldots ,X_{B_n},X_C)'=0$ switches stability at $R_0=1$. We formulate this as

#### **Theorem 1**

$R_0$, defined as the dominant eigenvalue of $T(-\varSigma )^{-1}$, is a threshold parameter with threshold value one for the zero state of (35).

Note that $T(-\varSigma )^{-1}$ is an $(n+2)\times (n+2)$ matrix. Also, elements $(T(-\varSigma )^{-1})_{xy}$ can be interpreted as the expected number of binding sites in $x$ created by one binding site in $y$, where $x,y\in \{A,B_1,\ldots ,B_n,C\}$. This gives us an interpretation of $R_0$ in terms of binding sites $A,B_1,\ldots ,B_n,C$. However, we can reduce the characterization of $R_0$ to a problem involving a $3\times 3$ matrix by averaging the $B_j$ in the right way (and this allows us to consider binding sites in $A$, ${\varvec{B}}$, $C$ only). We show this in the remainder of this subsection.

Consider the $3\times 3$ matrix $K=(k_{x,y})$ where the $k_{x,y}$, $x,y=A,{{\varvec{B}}},C$, are defined by

36 $$\begin{aligned} T(-\varSigma )^{-1}\psi _y=\sum _{x=A,{{\varvec{B}}},C}k_{x,y}\psi _x. \end{aligned}$$

Then $R_0$ is also the dominant eigenvalue of $K$. We formulate this in a theorem.

#### **Theorem 2**

$R_0$, defined as the dominant eigenvalue of $K$, where $K$ is defined by (36), is a threshold parameter with threshold value one for the zero state of (35).

#### *Proof*

We have defined $R_0$ as the dominant eigenvalue of $T(-\varSigma ^{-1})$ and this $R_0$ is a threshold parameter of the linear system corresponding to the matrix $T+\varSigma$ according to Theorem 1. We will show that $T(-\varSigma ^{-1})$ and $K$ have the same dominant eigenvalue.

The range of $T$ is spanned by three linearly independent vectors $\psi _A$, $\psi _{{\varvec{B}}}$, $\psi _C$. If $T(-\varSigma )^{-1}v=\lambda v$, with $\lambda \ne 0$, $v\ne 0$, then $v$ lies in the range of $T$, i.e. $v=\sum _{y}w_y\psi _y$, with at least one of the $w_y\ne 0$. Therefore,

$$\begin{aligned} T(-\varSigma )^{-1}v&=T(-\varSigma )^{-1}\left( \sum _{y}w_y\psi _y\right) \\&=\sum _{x}\sum _{y}k_{x,y}w_y\psi _x, \end{aligned}$$

where the summation is over $x$ or $y\in \{A,{\varvec{B}}, C\}$. On the other hand, this is equal to

$$\begin{aligned} \lambda v=\lambda \sum _x w_x\psi _x. \end{aligned}$$

Since the $\psi _x$ are linearly independent, it follows that

$$\begin{aligned} \sum _yk_{x,y}w_y=\lambda w_x, \end{aligned}$$

for all $x=A,{\varvec{B}},C$. In matrix notation:

$$\begin{aligned} Kw=\lambda w, \end{aligned}$$

where $w=(w_x)$ is a three-dimensional vector, not equal to the zero vector. We conclude that if $\lambda$ is a nonzero eigenvalue of $T(-\varSigma )^{-1}$, then $\lambda$ is also a nonzero eigenvalue of $K$. To find the dominant eigenvalue $R_0$ of $T(-\varSigma )^{-1}$, we can focus on the $3\times 3$ matrix $K=(k_{x,y})$. $\square$

Consider the definition of $K$ given by (36). This definition allows for an interpretation of the elements $k_{x,y}$. Indeed, $k_{x,y}$ can be interpreted as the expected number of binding sites in $x$ created by one binding site in $y$, with $x,y \in \{ A,{{\varvec{B}}},C\}$. Therefore, we call $K$ the NGM on the level of binding sites, and $R_0$ can be interpreted as the expected number of secondary cases caused by a typical newly infected binding site in the beginning of an epidemic. Note that when $x$ or $y$ equals ${\varvec{B}}$ we specify a probability distribution rather than a specific state.

The relation (36) completely characterizes the matrix $K$. However, using the interpretation, we can give explicit expressions for the entries of $K$; see Appendix C. In this appendix it is also shown that, in order to find $R_0$, we can reduce $K$ to a $2\times 2$ matrix and calculate the dominant eigenvalue of this smaller matrix. By combining (55)–(57), (59), and (61)–(63) we then find $R_0$ given as an explicit function of the model parameters.

We have characterized $R_0$ in terms of binding sites, both by considering all possible states $\{A,B_1,\ldots , B_n, C\}$ and by considering $\{A,{\varvec{B}}, C\}$. This allows for an interpretation of $R_0$ in terms of binding sites. As we next show, we may also interpret $R_0$ in terms of individuals.

### $R_0$ in terms of individuals

The model description is on the level of individuals, so it is only sensible that, in this section, we concern ourselves with the interpretation of $R_0$ in terms of individuals, i.e. the interpretation of $R_0$ as the expected number of secondary cases caused by a typical newly infected *individual* (rather than binding site) in the beginning of an epidemic.

Individuals can be considered as collections of $n$ binding sites. We find the relation between the binding site level and the individual level as follows. Recall (33), where we see in the second equality that the $\phi _j$ are a linear combination of the $\psi _A$, $\psi _{{\varvec{B}}}$, and $\psi _C$. Note that $\phi _j$ is a collection of $n$ infectious binding sites, $n-j$ in state $A$, 1 in state $C$, and $j-1$ in states $B_l$, $l=0,\ldots ,n$ (and where the infectious binding site is in state $B_l$ with probability $Q_l$). We can identify $\phi _j$ with an individual in state $(+,j-1,1)$. Note that the $(+,j-1,1)$ are the possible states of an individual at epidemiological birth. For the case $n=1$, we have $\phi _1=\psi _C$ only (which corresponds to the only state-at-epi-birth $(+,0,1)$ since an infectious individual at epi-birth is in a partnership with its epidemiological partner).

This observation allows us to also give an interpretation to $R_0$ for individuals. Indeed, consider $K^\mathrm{ind}=((k^\mathrm{ind})_{ij})$, where the $(k^\mathrm{ind})_{ij}$ are characterized by

37 $$\begin{aligned} T(-\varSigma ^{-1})\phi _j=\sum _{i=1}^n(k^\mathrm{ind})_{ij}\phi _i. \end{aligned}$$

Element $(k^\mathrm{ind})_{ij}$ is then the expected number of secondary cases in state i caused by one infectious individual in state $j$. Here i and $j$ are of the form $(+,m,1)$, $m=0,\ldots ,n-1$. To arrive at the interpretation of $R_0$ on the individual-level, we can prove that the dominant eigenvalue of $K^\mathrm{ind}$ (which is the NGM on individual level) equals the dominant eigenvalue $R_0$ of $K$; see Appendix E for the details.

The matrix $K^\mathrm{ind}$ is completely characterized by the identity (37). But, as in the case of $K$, we can use the interpretation to give a more explicit expression for the entries of $K^\mathrm{ind}$; see Appendix F.

### $R_0$: equivalence of different interpretations

In Sect. 5.3 $R_0$ is defined as the dominant eigenvalue of $T(-\varSigma )^{-1}$. Theorem 2 states that $R_0$ is also the dominant eigenvalue of the $ABC$-NGM $K$, where $K$ is defined by (36), and in Appendix C we show that, in order to find the dominant eigenvalue of $K$, we can reduce $K$ to a $2\times 2$ matrix $\tilde{K}$. Finally, in Appendix E, we show that $R_0$ is also the dominant eigenvalue of the NGM $K^\mathrm{ind}$ on individual level. We summarize this in (38), where $\Leftrightarrow$ refers to ‘has the same dominant eigenvalue’.

38 $$\begin{aligned} T(-\varSigma ^{-1})\quad \Longleftrightarrow&\quad K \quad \Longleftrightarrow \quad K^{\text {ind}}\\&\quad \Updownarrow \nonumber \\&\quad \tilde{K}\nonumber \end{aligned}$$

In the next section we prove that $R_0$ defined in this way is indeed a threshold for the stability of the disease-free steady state of the nonlinear system (18), by using $L$ defined in (27) to relate the linearisation of (18) to (35).

## Proof that $R_0$ is a threshold parameter

Recall that, using the mean field at distance one assumption, we have written down a system of differential equations to describe the transmission of the infectious disease across the dynamic network. We will refer to the system (18) of differential equations for the fractions of the population of individuals in states $\ell$, $\ell =(\pm ,k_-,k_+)$ as the $P$-system. In Sect. 4 we have linearised this system around the disease-free steady state and we were able to restrict this linearised system to the fractions $P_{(-,k,1)}$ and $P_{(+,k_-,k_+)}$. In Sect. 5 we considered binding sites of an infectious individual (in the linearisation!) and these binding sites could be in $A$, ${\varvec{B}}$, and $C$. This led to the $ABC$-system (35). $R_0$, defined as the dominant eigenvalue of $K$, is a threshold for the stability of the zero state of (35); this was formulated in Theorem 2. In this section we will prove that $R_0$ is also a threshold for the stability of the disease-free steady state of system (18). We do so by relating the reduced linearisation (26) of the $P$-system to the $ABC$-system (35).

### The case $n=1$

For $n=1$ the proof is relatively easy, since there is no distinction between ‘individual’ and ‘binding site’. As the proof provides guiding lines for the general case, we present it first.

If we write out (26) for $n=1$ we obtain the system of four ODE:

$$\begin{aligned} \frac{dP_{(-,0,1)}}{dt}&=-(\sigma +2\mu +\beta )P_{(-,0,1)}+\rho F_+ P_0 \\ \frac{dP_{(+,0,0)}}{dt}&=-(\rho \bar{F}+\mu )P_{(+,0,0)}+(\sigma +\mu )P_{(+,1,0)}+(\sigma +\mu )P_{(+,0,1)}\\ \frac{dP_{(+,1,0)}}{dt}&=-(\sigma +2\mu +\beta )P_{(+,1,0)}+\rho \bar{F}P_{(+,0,0)}\\ \frac{dP_{(+,0,1)}}{dt}&=-(\sigma +2\mu +\beta )P_{(+,0,1)}+\beta P_{(+,1,0)}+\beta P_{(-,0,1)}. \end{aligned}$$

The consistency relation (19), which for $n=1$ reduces to

39 $$\begin{aligned} P(-,0,1) = P(+,1,0), \end{aligned}$$

is reflected in the fact that the first and third equation of the system of ODEs are identical (recall that, for $n=1$, $\bar{F}$ equals $P_0$ and $F_+$ equals $P_{(+,0,0)}$). Using (39) we reduce to the three-dimensional system

$$\begin{aligned} \frac{dP_{(+,0,0)}}{dt}&=-(\rho \bar{F}+\mu )P_{(+,0,0)}+(\sigma +\mu )P_{(+,1,0)}+(\sigma +\mu )P_{(+,0,1)}\\ \frac{dP_{(+,1,0)}}{dt}&=-(\sigma +2\mu +\beta )P_{(+,1,0)}+\rho \bar{F}P_{(+,0,0)}\\ \frac{dP_{(+,0,1)}}{dt}&=-(\sigma +2\mu +\beta )P_{(+,0,1)}+2\beta P_{(+,1,0)}+\beta P_{(-,0,1)}. \end{aligned}$$

To finish the proof, we only need to observe that the corresponding matrix is exactly $\varSigma _1 + T_1$, with $\varSigma _1$ defined in (28) and $T_1$ in (29).

Indeed, recall the three states $A$, $B$, and $C$ that we defined for the binding site of an infectious individual in Sect. 5.1 and the population level fractions $X_A$, $X_B$, $X_C$ in states $A$, $B$, and $C$. Since individuals have exactly one binding site we identify the fractions of binding sites with fractions of individuals:

$$\begin{aligned} \begin{aligned} X_A&=P_{(+,0,0)}\\ X_B&=P_{(+,1,0)}\\ X_C&=P_{(+,0,1)}. \end{aligned} \end{aligned}$$

With this identification, the linearisation of the $P$-system equals the (linear) $ABC$-system. Therefore, not only is there a stability switch of the disease-free state of the $ABC$-system at $R_0=1$ (see also Theorem 2), but in fact there is also a stability switch for the disease-free state of the system at $R_0=1$.

**Fig. 6:**
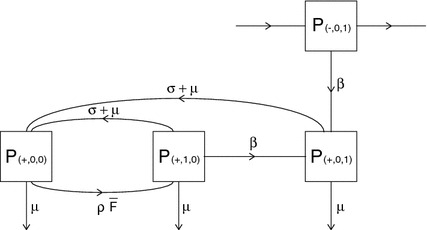
Flow chart representing part of the linearised system of ODEs (26) for the p-level fractions

To enhance the understanding, we present the main ingredients of the proof once more, but now by way of pictures. Figure 3 depicts the possible states and state transitions for an *infectious* individual. The corresponding part of the transition matrix is $\varSigma _1$. The corresponding p-level variables are $P_\ell$ with indices $\ell =(+,0,0), (+,1,0), (+,0,1)$. This $+$ part of the $P$-vector does not form a closed system. Indeed, an individual in state $(-,0,1)$ has probability per unit of time $\beta$ to jump to $(+,0,1)$, as indicated in Fig. 6.

**Fig. 7:**
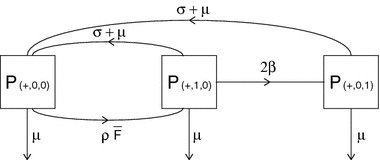
Flow chart of Fig. 6 with $P_{(-,0,1)}$ eliminated. Note that this figure does not allow for an individual-level interpretation; the rate at which an individual in state $(+,1,0)$ infects its susceptible partner is $\beta$ (compare with Fig. 3). But the flow from population-level fraction $P_{(+,1,0)}$ to $P_{(+,0,1)}$ is with rate $2\beta$ since it implicitly captures the inflow from $P_{(-,0,1)}$

When this jump occurs, the responsible partner (the ‘epidemiological parent’) jumps from $(+,1,0)$ to $(+,0,1)$. The interpretation underlying this last statement is mathematically reflected in the consistency relation (39). Using (39) we reduce the flow chart of Fig. 6 to the one depicted in Fig. 7. The corresponding matrix is $\varSigma _1+T_1$.

### Generalization: $n>1$

In general, for $n>1$, we can express $X_A$, $X_{B_j}$, and $X_C$ in terms of the linearised $P$-system by:

$$\begin{aligned} X_A&= \sum _{k_+=0}^n\sum _{k_-=0}^{n-k_+}(n-k_--k_+)P_{(+,k_-,k_+)}\\ X_{B_j}&= P_{(-,j-1,1)}\\ X_C&= \sum _{k_+=0}^n\sum _{k_-=0}^{n-k_+}k_+P_{(+,k_-,k_+)}. \end{aligned}$$

The explanation is as follows. An individual in state $(+,k_-,k_+)$ is infectious and has $n-k_{-}-k_{+}$ free binding sites and $k_+$ binding sites occupied by infectious partners. Summing over all possible states $(+,k_-,k_+)$ we obtain the number of binding sites in, respectively, states $A$ and $C$. For the number of binding sites in state $B_j$ we observe that an individual in $(-,j-1,1)$ has $j$ partners in total and one infectious partner. This infectious individual therefore has a binding site occupied by a susceptible partner who has $j$ partners in total, i.e. a binding site in state $B_j$. The total number of binding sites in state $B_j$ is therefore $P_{(-,j-1,1)}$.

So the map $L$ defined in (27) maps the $P$-variables to the $X_{ABC}$-variables, i.e. we have the linear transformation

40 $$\begin{aligned} \begin{pmatrix}X_A\\ X_{B_1}\\ \vdots \\ X_{B_n}\\ X_C\end{pmatrix}=LP. \end{aligned}$$

By differentiating $LP$ and using (26), we obtain the linear system of differential equations (35) for $X_A$, $X_{B_j}$, and $X_C$.

It remains to prove that the stability switch of the zero state of the $ABC$-system occurs if and only if the disease-free state of the $P$-system (18) switches stability. This will be shown in the remainder of this section.

We know that $R_0$ is a threshold parameter for the zero state of the $ABC$-system (see Theorem 2), i.e.

41 $$\begin{aligned} {{\mathrm{sign}}}(R_0-1)={{\mathrm{sign}}}(r_{ABC}), \end{aligned}$$

where $r_{ABC}$ is the spectral bound of $T+\varSigma$, i.e. $r_{ABC}=\sup \{\text {Re}(\lambda ) :\lambda \in \sigma (T+\varSigma )\}$, and $\sigma (T+\varSigma )$ is the spectrum of $T+\varSigma$.

So in order to show that $R_0$ is a threshold for the disease free state of the $P$-system, it suffices to show that

42 $$\begin{aligned} {{\mathrm{sign}}}(r_{ABC})={{\mathrm{sign}}}(r_P). \end{aligned}$$

Here $r_P$ is the spectral bound of $M_P$ where $M_P$ is the matrix corresponding to the right-hand side of (26). In fact we will show that $r_{ABC}=r_P$.

We will proceed as follows. First we shall prove that $r_{ABC}$ and $r_P$ are dominant eigenvalues of the matrices $T+\varSigma$ and $M_P$, respectively, in the sense that these eigenvalues are uniquely characterized by the positivity of the eigenvector (up to a multiplicative positive constant).

We show in Lemmas 4 and 5 that $T+\varSigma$ and $M_P$ are irreducible matrices. This then allows us to conclude that the dominant eigenvalues of $M_P$ and $T+\varSigma$ are real and uniquely characterized by a positive eigenvector (see e.g. Theorem 2.5 of Seneta 1973). In other words, there exists a real eigenvalue $r_P$ for $M_P$ for which it holds that $r_P>\text {Re } \lambda$ for any eigenvalue $\lambda \ne r_P$ of $M_P$ and $r_P$ is uniquely defined by the positivity of the corresponding eigenvector (and similarly with $r_{ABC}$ replacing $r_P$ and $T+\varSigma$ replacing $M_P$).

In Lemmas 4 and 5 below we use that a matrix $M=(m_{xy})$ is irreducible if and only if variable $x$ communicates with variable $y$ ($x\leftrightarrow y$) for all variables $x$ and $y$, i.e. there is a path from $x$ to $y$ ($x\rightarrow y$), i.e. there are variables $y_1$, $y_2$, $\ldots$, $y_n$ such that $m_{y,y_n}\cdots m_{y_2,y_1}m_{y_1,x}>0$, and a path from $y$ to $x$ ($y\rightarrow x$), i.e. there are variables $x_1$, $x_2$, $\ldots$, $x_k$ such that $m_{y,x_k}\cdots m_{x_2,x_1}m_{x_1,y}>0$. Note that the somewhat unusual notation is due to our convention that $m_{xy}$ denotes the transition from $y$ to $x$ (instead of the transition from $x$ to $y$, as it is common in the stochastic community).

#### **Lemma 4**

$T+\varSigma$ is an irreducible matrix.

#### *Proof*

The flowchart describing the matrix $\varSigma$ is presented in Fig. 4. We immediately see from this figure that from any state $x$ there is a path to any other state $y$, with $x,y\in \{A,B_1,B_2,\ldots ,B_n,C\}$. It follows that $\varSigma$ is irreducible. Since $T$ is nonnegative, also $T+\varSigma$ is irreducible. $\square$

#### **Lemma 5**

$M_{P}$ is an irreducible matrix.

#### *Proof*

With respect to a splitting of $P$ into $-$ components and $+$ components, $M_P$ is a block matrix that consists of four matrices $M_1$, $M_2$, $M_3$, $M_4$:The matrices $M_2$ and $M_3$ are non-negative matrices, not equal to the zero matrix, while $M_1$ and $M_4$ are positive off-diagonal. We show that $M_1$ and $M_4$ are irreducible, and that this implies that $M_P$ is irreducible.

**Fig. 8:**
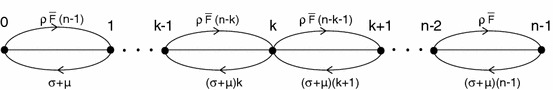
Graphical representation of part of matrix $M_1$ (point $k$ represents fraction $P_{(-,k,1)}$) showing that $x\rightarrow y$ for all $x,y=P_{(-,l,1)}$, i.e. $M_1$ is irreducible. Part of $M_1$ that is being ignored is e.g. the rates $\beta +\sigma +2\mu$ out of each variable $P_{(-,k_-,1)}$ leaving the $-$ system

Consider $M_1$. This matrix consists of the rates corresponding to the possible flows of the $-$ variables, i.e. population-level fractions of the form $P_{(-,k,1)}$ (and rates $\beta +\sigma +2\mu$ out of the $-$ states, that we do not need to consider here). In Fig. 8 part of the possible flows and corresponding rates are represented graphically. From Fig. 8 we immediately see that, from any variable $P_{(-,k,1)}$, one can find a path to any other variable $P_{(-,l,1)}$, or in other words, $P_{(-,k,1)}\rightarrow P_{(-,l,1)}$ for all $k,l=0,\ldots ,n-1$. Therefore $M_1$ is an irreducible matrix.

**Fig. 9:**
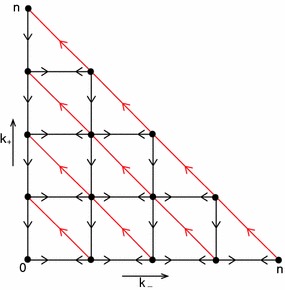
Graphical representation of the possible flows incorporated in the matrix $M_4$ (coordinate $(k_-,k_+)$ represents fraction $P_{(+,k_-,k_+)}$), ignoring the death rate $\mu$ out of each fraction

**Fig. 10:**
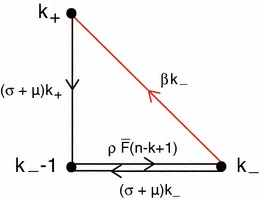
Rates corresponding to the flows of Fig. 9, ignoring the death rate $\mu$ out of each variable

Consider the matrix $M_4$. This matrix consists of the rates corresponding to the possible flows of the $+$ variables. i.e. population-level fractions of the form $P_{(+,k_-,k_+)}$. In Fig. 9 a graphical representation of part of the possible flows are given and in Fig. 10 the rates corresponding to these flows are given. These figures show (literally) that $M_4$ is irreducible.

Finally, consider two variables $x^-=P_{(-,k,1)}$, $x^+=P_{(-,l_-,l_+)}$ of the matrix $M_P$. We show that $x^-\leftrightarrow x^+$.

Since $M_2$ and $M_3$ are non-negative and non-zero, there are variables $y^-$, $y^+$, $z^-$, $z^+$ such that $y^-\rightarrow y^+$ and $z^+\rightarrow z^-$. Note that, in terms of interpretation, the nonzero elements of $M_2$ correspond to infection of $-$ individuals by one of their $+$ partner, i.e. transitions with rate $\beta$ from fractions $P_{(-,j_-,1)}$ to $P_{(+,j,1)}$. The nonzero elements of $M_3$ correspond to the feed into the $P_{(-,j_-,1)}$ category via the $F_+$ terms from fractions $P_{(+,k_-,k_+)}$.

We find a path from $x^-\rightarrow x^+$ through $y^-$ and $y^+$, i.e.

$$\begin{aligned} x^-\rightarrow y^-\rightarrow y^+\rightarrow x^+, \end{aligned}$$

and a path from $x^+\rightarrow x^-$ through $z^+$ and $z^-$, i.e.

$$\begin{aligned} x^+\rightarrow z^+\rightarrow z^-\rightarrow x^-. \end{aligned}$$

Note that the paths $x^-\rightarrow y^-$, $y^+\rightarrow x^+$, $x^+\rightarrow z^+$, and $z^-\rightarrow x^-$ exist since $M_1$ and $M_4$ are irreducible.

Since any two variables $x^-$ and $x^+$ of $M_P$ communicate, i.e. $x^-{{\mathrm{\leftrightarrow }}}x^+$, $M_P$ is irreducible. $\square$

We now have all the ingredients to prove that $R_0$ is a threshold parameter for the disease free state of (18).

Since $M_P$ is an irreducible positive off-diagonal matrix, we know that $M_P$ has a real dominant eigenvalue $r_P$ with corresponding positive eigenvector $v$, i.e.

$$\begin{aligned} M_P\, v=r_P\, v. \end{aligned}$$

Then how does this relate to $T+\varSigma$? On the one hand we find that

$$\begin{aligned} \frac{d}{dt}LP=L\frac{dP}{dt}=LM_PP, \end{aligned}$$

on the other hand $LP=x$ and (35) holds. Therefore

43 $$\begin{aligned} LM_PP=(T+\varSigma )LP, \end{aligned}$$

and it follows that

$$\begin{aligned} r_PLv=LM_P\, v=(T+\varSigma )Lv. \end{aligned}$$

We have seen in Sect. 4 that if $v$ is strictly positive then so is $Lv$. Furthermore, since $T+\varSigma$ is an irreducible positive off-diagonal matrix (see Lemma 4), the Malthusian parameter of $T+\varSigma$ is uniquely characterized by a positive eigenvector. Therefore $r_P$ is also the Malthusian parameter of $T+\varSigma$ with corresponding eigenvector $Lv$, i.e.

44 $$\begin{aligned} r_P=r_{ABC}. \end{aligned}$$

Finally, (42) together with Theorem 2 shows that $R_0$ is a threshold parameter of the $P$-system.

### Characterization of the Malthusian parameter $r$ ($=r_{ABC}=r_P$)

In this section we characterize the initial exponential growth rate $r=r_{ABC}=r_P$ (recall (44)). The Malthusian parameter $r$ satisfies

$$\begin{aligned} (T+\varSigma )v=rv\quad \Leftrightarrow \quad Tv=(rI-\varSigma )v. \end{aligned}$$

So $(rI-\varSigma )v$ lies in the range of $T$, i.e.

$$\begin{aligned} (rI-\varSigma )v=w \end{aligned}$$

with

45 $$\begin{aligned} w=d_A\psi _A+d_B\psi _{{\varvec{B}}}+d_C\psi _C, \end{aligned}$$

where the $d_x$ are some constants, not all equal to zero, and the $\psi _x$ are defined in (34), $x=A,{\varvec{B}},C$. This is equivalent to

$$\begin{aligned} v=(rI-\varSigma )^{-1}w. \end{aligned}$$

Therefore

$$\begin{aligned} T(rI-\varSigma )^{-1}w=w, \end{aligned}$$

where $w$ is defined by (45), so

46 $$\begin{aligned} \sum _xd_xT(rI-\varSigma )^{-1}\psi _x=\sum _xd_x\psi _x. \end{aligned}$$

Since the range of $T$ is spanned by $\psi _x$, we also have that, for certain constants $(m_r)_{yx}$,

47 $$\begin{aligned} T(rI-\varSigma )^{-1}\psi _x=\sum _{y}(m_r)_{yx}\psi _y. \end{aligned}$$

The Malthusian parameter $r$ then needs to satisfy

$$\begin{aligned} M_rd=d, \end{aligned}$$

where $M_r=((m_r)_{xy})$ is a $3\times 3$ matrix characterized by (47), with matrix elements $(m_r)_{xy}$ depending on the unknown $r$. Identity (47) fully characterizes elements $(m_r)_{xy}$, but, as in the case of $K$ and $K^{\text {ind}}$, we can use the interpretation to give explicit expressions for the entries of $M_r$, in the last paragraph of Appendix C we outline how this can be done.

Finally, consider the case $n=1$, then $r$ satisfies

48 $$\begin{aligned} T_1(rI-\varSigma _1)^{-1}w=w, \end{aligned}$$

where $\varSigma _1$ and $T_1$ are defined in (28) and (29), respectively. Since the range of $T_1$ is spanned by $\psi _C$, we see that $w=\psi _C$. We find that

$$\begin{aligned} T_1(rI-\varSigma _1)^{-1}\begin{pmatrix}0\\ 0\\ 1\end{pmatrix}=\begin{pmatrix}0\\ 0\\ \frac{\beta \rho \bar{F}(\sigma +\mu )}{(r+\mu )(r+\beta +\sigma +2\mu )(r+\rho \bar{F}+\sigma +2\mu )}\end{pmatrix}. \end{aligned}$$

It then follows from (48) that we find $r$ by solving the following third-order polynomial in $r$:

$$\begin{aligned} \beta \rho \bar{F}(\sigma +\mu )=(r+\mu )(r+\beta +\sigma +2\mu )(r+\rho \bar{F}+\sigma +2\mu ). \end{aligned}$$

## Looking back and ahead

The overall aim of our research is to formulate and analyse models for the spread of an infectious disease across a network that is dynamic in the double sense that individuals come (by birth) and go (by death) and that links/partnerships are formed and broken. In particular our aim is to investigate the role of concurrency in the spread of sexually transmitted infections.

In Leung et al. (2012) we introduced a class of doubly dynamic network models that are relatively simple to describe, that involve just a few parameters, and for which one can calculate many statistics exactly in explicit detail. The next step, taken here, is superimposing the spread of an infection. In order to retain the simplicity, we again characterize individuals by their dynamic degree (i.e. the current number of their partners), but now include the disease status ($S$ versus $I$) of the individual itself and of its partners. In this bookkeeping scheme we need to account for the infection of a partner by one of its other partners, but the scheme itself does not provide information about partners of partners. Thus we faced a closing problem. The mean field at distance one assumption provided a natural solution.

Originally we thought that this was an assumption because we had not yet found a way to prove it. In a late stage Pieter Trapman pointed the way to the current Appendix B, showing that the assumption is inconsistent with the model itself. We then realised that, in essence, our bookkeeping scheme constitutes a first order description that we close by making the (inconsistent) mean field at distance one assumption. So the deterministic system studied here provides at best an approximation to the large system size limit of a stochastic model.

The great advantage of the deterministic system of dimension $(n+1)(n+2)$ is that it is amenable to analysis. The fact that binding sites operate to some extent independently from each other enables a reduction of the dimension from $(n+1)(n+2)$ to $2$ in the characterization of $R_0$. Indeed, we characterized the basic reproduction number $R_0$ as the dominant eigenvalue of a $3\times 3$ matrix with elements describing the expected numbers of newly infected binding sites of three different types generated by one infected binding site of either type during its life time. We could then further reduce the $3\times 3$ matrix to a $2\times 2$ matrix which lead to an explicit expression for the dominant eigenvalue $R_0$. We also verified that the basic reproduction number $R_0$ defined in this way is indeed a threshold parameter for the stability of the disease free steady state of the nonlinear system of model equations. This is done by establishing a relationship between the exponential growth rate $r$ of the epidemic in the linearised system and the quantity $R_0$ on the level of binding sites.

The characterization of $r$ and $R_0$ opens up the route for investigating the impact of concurrency on the transmission of the ${ SI}$ infection in the dynamic network. We can now study how $r$ and $R_0$ depend on the capacity $n$ when fixing all other parameters at constant values. Furthermore, the relationship between concurrency measures on the one hand and $R_0$, $r$, and the endemic steady state on the other, can be analysed. This will be explored in a follow-up paper. (Concerning the endemic steady state, we will need to derive the equations that characterize it, to investigate the uniqueness and to prove that existence requires $R_0>1$.)

There are a number of generalisations of the network model that are both useful and feasible. The extension to a heterosexual population requires only the distinction between males and females and some assumptions on the symmetry or asymmetry in rates and partnership capacity between the two sexes. We expect that all results presented here carry, mutatis mutandis, over to that situation. No doubt the model can also be extended to the situation that $n$ is a random variable with a prescribed distribution.

Other generalisations pertain to the description of infectiousness. An obvious example is a model with two consecutive stages $I_1$ and $I_2$, where infectiousness is characterised by $\beta _i$ in stage $I_i$. Other compartmental epidemic models could be considered as well, such as ${ SIR}$ and ${ SIS}$. Inclusion of the impact of the disease on mortality is very relevant in the context of HIV. Unfortunately it might turn out to be very hard.

The most stringent limitation of our framework is the assumption that having a partner does not influence an individual’s propensity to enter into a new partnership or its contact rate in other ongoing partnerships. This is clearly at odds with reality (although equally clearly it is an impossible task to disentangle the manifold ways in which dependence ‘works’ in reality). Dependence destroys the basis on which our analytic approach rests.

Be that as it may, we view the work presented here as a first step towards a framework for studying the impact of dynamic network structure on the transmission of an infectious disease.

## Figures and Tables

**Fig. 11:**
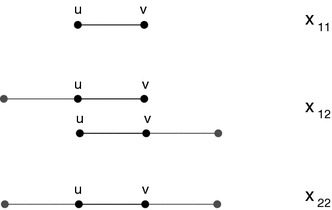
The three possible configurations for the partnership $uv$ concerning additional partners when $n=2$

**Fig. 12:**
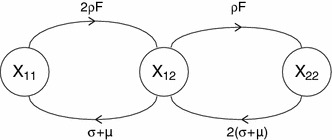
The flowchart for the three possible configurations for the partnership $uv$ concerning additional partners given that $uv$ exists for the period under consideration

**Fig. 13:**
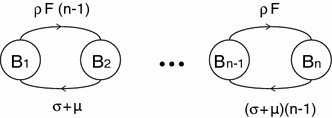
State transitions and corresponding rates between states $B_1,\ldots ,B_n$; the corresponding transition matrix is denoted $M$

**Fig. 14:**
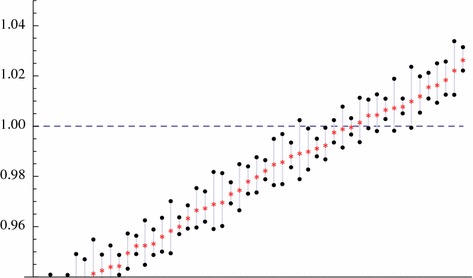
For different values of parameters $\rho$, $\sigma$, $\mu$, $\beta$, the basic reproduction number $R_0$ is determined together with a lower and an upper bound for ‘the real $R_0$’. We focus here on parameter values for which $R_0$ lies around threshold value 1

## Appendix A: $R_0$ for $n=1$: alternative method

We can also characterize $R_0$ for the case $n=1$ in the following way. An individual at epidemiological birth is in state $C$ with probability one (since it has its epidemiological parent as partner) and each time it visits $C$ (which is only possible by jumping from $B$ to $C$ since the probability to encounter an infectious individual is zero at the beginning of an epidemic) a new infectious individual is created; recall Fig. 3. So, for $R_0$, we simply need to count the expected number of times to visit $C$ when starting in $C$. This can be done by exploiting the Markov property:

49 $$\begin{aligned} R_0=\pi _{CC}(1+R_0), \end{aligned}$$

where $\pi _{CC}$ is the probability to ever enter state $C$ when starting in state $C$. Let $\pi _{xy}$ denote the probability to ever enter state $x$ when starting in $y$, $x,y\in \{A,B,C\}$. We find $\pi _{CC}$ by first-step analysis:

$$\begin{aligned} \pi _{CC}&=\frac{\sigma +\mu }{\sigma +2\mu }\pi _{CA}\\ \pi _{CA}&=\frac{\rho \bar{F}}{\rho \bar{F}+\mu }\pi _{CB}\\ \pi _{CB}&=\frac{\beta }{\beta +\sigma +2\mu }+\frac{\sigma +\mu }{\beta +\sigma +2\mu }\pi _{CA}. \end{aligned}$$

Solving for $\pi _{CC}$ we find

$$\begin{aligned} \pi _{CC}=\frac{\beta \rho \bar{F}(\sigma +\mu )}{(\sigma +2\mu )\left( \rho \bar{F}(\beta +\mu )+\mu (\beta +\sigma +2\mu )\right) }. \end{aligned}$$

This yields the same expression (30) as the $ABC$ scheme does.

## Appendix B: Correlation between the states of two partners

Consider a randomly chosen partnership. For convenience we call the individuals in the partnership $u$ and $v$. Then, without knowing anything about $v$, the probability that $u$ is in state $k$, $k\ge 1$, is given by $Q_k$ (Lemma 2), i.e.

$$\begin{aligned} P(u \text { in state }k)=Q_k. \end{aligned}$$

In other words, $Q_k$ is the probability that an individual is in state $k$ given that it has at least one partner.

Let’s study this partnership in more detail. The states of $u$ and $v$ are independent of each other at the moment $t=0$ when the partnership $uv$ is formed, i.e.

$$\begin{aligned}&P(u\text { in state }k\text { and }v\text { in state }l\text { at }t=0)\\&\quad =P(u\text { in state }k\text { at }t=0)P(v\text { in state }l \text { at }t=0)\\&\quad =q_kq_l, \end{aligned}$$

cf. assumption (7).

As long as we condition on the existence of the partnership $uv$ the remaining binding sites of $u$ behave independently of the remaining binding sites of $v$ and consequently there is independence of the states of $u$ and $v$ at any time $t=s$ in the partnership, i.e.

$$\begin{aligned}&P(u\text { in state }k\text { and }v\text { in state }l\text { at }t=s)\\&\quad =P(u \text { in state }k\text { at }t=s)P(v\text { in state }l\text { at }t=s). \end{aligned}$$

However, if we do *not* specify the duration so far of the partnership, then we find dependence between the states of $u$ and $v$:

$$\begin{aligned}&P(u\text { in state }k\text { and }v\text { in state }l)\\&\quad =\int _0^\infty (\sigma +2\mu )e^{-(\sigma +2\mu )s}P(u\text { in state }k\text { at }t=s\text { and }v\text { in state }l\text { at }t=s)ds\\&\quad =\int _0^\infty (\sigma +2\mu )e^{-(\sigma +2\mu )s}P(u\text { in state }k\text { at }t=s)P(v\text { in state }l\text { at }t=s)ds\\&\quad \ne P(u \text { in state }k)P(v\text { in state }l) =Q_kQ_l. \end{aligned}$$

Here the density $(\sigma +2\mu )e^{-(\sigma +2\mu )s}$ accounts for the conditioning on the $uv$ partnership remaining in existence. We show the inequality with explicit calculations for $n=2$. In this case, individuals can have 0, 1, or 2 partners. Choose a partnership at random from the population and label the partners $u$ and $v$. Since $n=2$, $u$ and $v$ both have one additional binding site that can be either free or occupied. This gives us three possible states for the partnership $uv$, we denote these states by $x_{11}$, $x_{12}$, and $x_{22}$; see Fig. 11.

Let $\pi (t)$ denote the probability distribution for the configuration of the partnership $uv$ at time $t$ given that $uv$ exists for the period under consideration.

At $t=0$ the probability distribution of the different configurations is given by

$$\begin{aligned} \pi (0)=\begin{pmatrix} q_1^2\\ 2q_1q_2\\ q_2^2 \end{pmatrix}. \end{aligned}$$

Given that the partnership $uv$ exists for the time interval under consideration, the transitions and the corresponding rates are represented by the flowchart in Fig. 12. We denote the corresponding transition matrix by $M$.

Note that the ‘age’ of the partnership $uv$ is exponentially distributed with parameter $\sigma +2\mu$. Therefore the probability distribution for the configuration of partnership $uv$ at the moment we pick the partnership from the pool of partnerships is

50 $$\begin{aligned} (\sigma +2\mu )\int _0^\infty e^{-t(\sigma +2\mu )I}\pi (t)dt&=(\sigma +2\mu )\int _0^\infty e^{t(M-(\sigma +2\mu )I)}\pi (0)dt\nonumber \\&=-(\sigma +2\mu )(M-(\sigma +2\mu )I)^{-1}\pi (0)\end{aligned}$$

51 $$\begin{aligned}&=\begin{pmatrix} Q_1^2+c\\ 2Q_1Q_2-2c\\ Q_2^2+c \end{pmatrix}\!, \end{aligned}$$

where

$$\begin{aligned} c=\frac{(\rho F\mu )^2}{(\sigma +2\mu )(2\rho F+3\sigma +4\mu )(2\rho F+2\sigma +3\mu )^2}. \end{aligned}$$

The states of $u$ and $v$ are independent of one another iff $c=0$

Note that $c=0$ if $\mu =0$ and $\mu =0$ corresponds to a dynamic network without demography. One should, however, not conclude that demography necessarily leads to correlation. We assumed that individuals are born single. One can think of other ways of incorporating demography, e.g. individuals having $k$ partners at birth with probability equal to the degree distribution (Kamp 2010). Adopting this rule creates a partnership network where, when disease is *not* considered, the correlation is zero between the degrees of two partners [in essence this rule makes ‘death’ the same as rewiring of partnerships after an exponentially distributed amount of time and then basically a dynamic network in a closed population is considered (Miller et al. 2012)].

## Appendix C: The matrix elements $k_{x,y}$ of $K$ and a characterization of $R_0$ by a $2\times 2$ matrix

In this appendix we use the interpretation to guide us in deriving explicit expressions for the $k_{x,y}$ and in that way we derive an explicit expression for $R_0$.

First of all, as explained below, the following equalities hold:

52 $$\begin{aligned} (-\varSigma )^{-1}\psi _A&=\left( \begin{array}{c}\frac{\sigma +2\mu }{\mu (\rho \bar{F}+\sigma +2\mu )}\ \frac{\rho \bar{F}(\sigma +2\mu )}{\mu (\rho \bar{F}+\sigma +2\mu )}(-\varSigma _{{\varvec{B}}})^{-1}q\\ \frac{\beta }{(\beta +\sigma +2\mu )(\sigma +2\mu )}\frac{\rho \bar{F}(\sigma +2\mu )}{\mu (\rho \bar{F}+\sigma +2\mu )}\end{array}\right) ,\end{aligned}$$

53 $$\begin{aligned} (-\varSigma )^{-1}\psi _{{\varvec{B}}}&=\begin{pmatrix}0\\ (-\varSigma _{{\varvec{B}}})^{-1}Q\\ 0\end{pmatrix}+\begin{pmatrix}\frac{\sigma +\mu }{\mu (\rho \bar{F}+\sigma +2\mu )}\\ \frac{\rho \bar{F}(\sigma +\mu )}{\mu (\rho \bar{F}+\sigma +2\mu )}(-\varSigma _{{\varvec{B}}})^{-1}q\\ \frac{\beta (\rho \bar{F}+\mu )}{\mu (\beta +\sigma +2\mu )(\rho \bar{F}+\sigma +2\mu )}\end{pmatrix}\!,\end{aligned}$$

54 $$\begin{aligned} (-\varSigma )^{-1}\psi _C&=\begin{pmatrix}\frac{\sigma +\mu }{\mu (\rho \bar{F}+\sigma +2\mu )}\\ \frac{\rho \bar{F}(\sigma +\mu )}{\mu (\rho \bar{F}+\sigma +2\mu )}(-\varSigma _{{\varvec{B}}})^{-1}q\\ \frac{\beta (\rho \bar{F}+\mu )}{\mu (\beta +\sigma +2\mu )(\rho \bar{F}+\sigma +2\mu )}+\frac{1}{\beta +\sigma +2\mu }\end{pmatrix}\!. \end{aligned}$$

Indeed, if we multiply the right-hand side of each of these equalities with the matrix $\varSigma$, where $\varSigma$ is defined in (31), we obtain $-\psi _x$, $x=A,{{\varvec{B}}},C$.

The elements $-\varSigma ^{-1}\psi _x$, $x=A,{{\varvec{B}}},C$, also have an interpretation. The interpretation of $(-\varSigma ^{-1}\psi _A)_{B_k}$ is as follows. Consider a binding site in state $A$. The probability for the binding site to be in state $A$ in the time interval $[0,\tau ]$ is

$$\begin{aligned} \left[ e^{\tau \varSigma _1}\begin{pmatrix}1\\ 0\\ 0\end{pmatrix}\right] _{A}, \end{aligned}$$

where $\varSigma _1$ is defined in (28). It enters the set ${\varvec{B}}$ at rate $\rho \bar{F}$. The probability that the binding site is in state $B_k$ upon entering ${\varvec{B}}$ is $q_k$. The probability to remain in $B_k$ in the time interval $[\tau ,s]$ is the $k$th component of

$$\begin{aligned} e^{(s-\tau )\varSigma _{{\varvec{B}}}}q. \end{aligned}$$

By integrating over all possible $s$ we find the expectation:

$$\begin{aligned}&\int _0^\infty \int _0^s\left[ e^{\tau \varSigma _1}\begin{pmatrix}1\\ 0\\ 0\end{pmatrix}\right] _{A}\rho \bar{F}e^{(s-\tau )\varSigma _{{\varvec{B}}}}q\,\mathrm {d}\tau \, \mathrm {d}s\\&\quad = \rho \bar{F}\left[ -\varSigma _1^{-1}\begin{pmatrix}1\\ 0\\ 0\end{pmatrix}\right] _{A}(-\varSigma _{{\varvec{B}}})^{-1}q\\&\quad = \frac{\rho \bar{F}(\sigma +2\mu )}{\mu (\rho \bar{F}+\sigma +2\mu )}(-\varSigma _{{\varvec{B}}})^{-1}q. \end{aligned}$$

The $k$th component is then the mean time a binding site that starts in state $A$ will spend in state $B_k$. Similarly, one can interpret $(-\varSigma ^{-1}\psi _C)_{B_k}$.

Finally, we show how to derive (53) by exploiting the interpretation of $-\varSigma ^{-1}\psi _{{\varvec{B}}}$ (this is the most complicated case and it involves all building blocks for the other cases).

First note that $(e^{s\varSigma }\psi _x)_y$ is the probability to be in state $y$ at time $s$ when one starts life in state $x$, so $((-\varSigma )^{-1}\psi _x)_y$, $x,y=A,{{\varvec{B}}},C$ is the mean time spent in state $y$ when the binding site starts its life in state $x$. Therefore

$$\begin{aligned} \int _0^\infty e^{s\varSigma }\psi _xds=(-\varSigma )^{-1}\psi _x, \end{aligned}$$

There are flows out of ${\varvec{B}}$ to $A$ and $C$ with rates $\sigma +\mu$ and $\beta$, respectively. There is also a flow in to ${\varvec{B}}$ from $A$ with rate $\rho \bar{F}$. Note that the transition matrix between state $A$, set ${\varvec{B}}$, and state $C$ is exactly $\varSigma _1$, where $\varSigma _1$ is defined in (28).

We shall use the inverse $(-\varSigma _1)^{-1}$ in the following calculations. Using linear algebra or interpretation, we obtain

$$\begin{aligned} (-\varSigma _1)^{-1}=\begin{pmatrix}\frac{\sigma +2\mu }{\mu (\rho \bar{F}+\sigma +2\mu )} &{} \frac{\sigma +\mu }{\mu (\rho \bar{F}+\sigma +2\mu )} &{} \frac{\sigma +\mu }{\mu (\rho \bar{F}+\sigma +2\mu )}\\ \frac{\rho \bar{F}(\sigma +2\mu )}{\mu (\beta +\sigma +2\mu )(\rho \bar{F}+\sigma +2\mu )} &{} \frac{(\rho \bar{F}+\mu )(\sigma +2\mu )}{\mu (\beta +\sigma +2\mu )(\rho \bar{F}+\sigma +2\mu )} &{} \frac{(\rho \bar{F}+\mu )(\sigma +2\mu )}{\mu (\beta +\sigma +\mu )(\rho \bar{F}+\sigma +2\mu )}\\ \frac{\beta \rho \bar{F}}{\mu (\beta +\sigma +2\mu )(\rho \bar{F}+\sigma +2\mu )} &{} \frac{\beta (\rho \bar{F}+\mu )}{\mu (\beta +\sigma +2\mu )(\rho \bar{F}+\sigma +2\mu )} &{} \frac{\beta (\rho \bar{F}+\mu )+\mu (\rho \bar{F}+\sigma +2\mu )}{\mu (\beta +\sigma +2\mu )(\rho \bar{F}+\sigma +2\mu )}\end{pmatrix}. \end{aligned}$$

The mean time a binding site born in one of the states $B_j$ in the set ${\varvec{B}}$, spends in state $A$ is given by

$$\begin{aligned} \left[ (-\varSigma _1)^{-1}\begin{pmatrix}0\\ 1\\ 0\end{pmatrix}\right] _A=\frac{\sigma +\mu }{\mu (\rho \bar{F}+\sigma +2\mu )}, \end{aligned}$$

while the mean future time a binding site presently in ${\varvec{B}}$ spends in state $C$ is

$$\begin{aligned} \left[ (-\varSigma _1)^{-1}\begin{pmatrix}0\\ 1\\ 0\end{pmatrix}\right] _C=\frac{\beta (\rho \bar{F}+\mu )}{\mu (\beta +\sigma +2\mu )(\rho \bar{F}+\sigma +2\mu )}. \end{aligned}$$

To determine the mean time spent in the set ${\varvec{B}}$, when starting life in ${\varvec{B}}$, is a bit more complicated. First of all, a binding site that starts life in ${\varvec{B}}$ starts life in state $B_j$ with probability $Q_j$, $j=1,\ldots ,n$. The mean time it then spends in state $B_k$ without leaving the set ${\varvec{B}}$ is given by

$$\begin{aligned} ((-\varSigma _{{\varvec{B}}})^{-1}Q)_k. \end{aligned}$$

Next, the binding site in ${\varvec{B}}$ leaves ${\varvec{B}}$ and enters $A$ with probability

$$\begin{aligned} \frac{\sigma +\mu }{\beta +\sigma +2\mu }. \end{aligned}$$

If that is the case, then the mean future time it spends in $B_k$ is, by first step analysis,

$$\begin{aligned} ((-\varSigma )^{-1}\psi _A)_{B_k}=\frac{\rho \bar{F}(\sigma +2\mu )}{\mu (\rho \bar{F}+\sigma +2\mu )}((-\varSigma _{{\varvec{B}}})^{-1}q)_{k}. \end{aligned}$$

Similarly, the probability to enter state $C$ from ${\varvec{B}}$ is

$$\begin{aligned} \frac{\beta }{\beta +\sigma +2\mu } \end{aligned}$$

and the mean time it will spend in $B_k$ is

$$\begin{aligned} ((-\varSigma )^{-1}\psi _C)_{B_j}=\frac{\rho \bar{F}(\sigma +\mu )}{\mu (\rho \bar{F}+\sigma +2\mu )}\left( (-\varSigma _{{\varvec{B}}})^{-1}q\right) _{j}. \end{aligned}$$

Therefore, the mean time spent in state $B_k$ after leaving ${\varvec{B}}$ is

$$\begin{aligned}&\frac{\sigma +\mu }{\beta +\sigma +2\mu }\frac{\rho \bar{F}(\sigma +2\mu )}{\mu (\rho \bar{F}+\sigma +2\mu )}[(-\varSigma _{{\varvec{B}}})^{-1}q]_{k}\\&\qquad +\frac{\beta }{\beta +\sigma +2\mu }\frac{\rho \bar{F}(\sigma +\mu )}{\mu (\rho \bar{F}+\sigma +2\mu )}[(-\varSigma _{{\varvec{B}}})^{-1}q]_{k}\\&\quad =\frac{\rho \bar{F}(\sigma +\mu )}{\mu (\rho \bar{F}+\sigma +2\mu )}((-\varSigma _{{\varvec{B}}})^{-1}q)_{k}. \end{aligned}$$

This explains (53).

The matrix $T$ is given by (32). By multiplying with (52), (53), and (54), we obtain the first column of $K$

55 $$\begin{aligned} \begin{pmatrix}k_{AA}\\ k_{{\varvec{B}}A}\\ k_{CA}\end{pmatrix}=\frac{\beta \rho \bar{F}(\sigma +2\mu )}{\mu (\rho \bar{F}+\sigma +2\mu )}\begin{pmatrix}\sum _{k=1}^n(n-k)[(-\varSigma _{{\varvec{B}}})^{-1}q]_k\\ \sum _{k=1}^n(k-1)[(-\varSigma _{{\varvec{B}}})^{-1}q]_k\\ \sum _{k=1}^n[(-\varSigma _{{\varvec{B}}})^{-1}q]_k\end{pmatrix}, \end{aligned}$$

the second column of $K$ is given by

56 $$\begin{aligned} \begin{pmatrix}k_{A{\varvec{B}}}\\ k_{{\varvec{B}}{\varvec{B}}}\\ k_{C{\varvec{B}}}\end{pmatrix}&= \beta \begin{pmatrix}\sum _{k=1}^n(n-k)[(-\varSigma _{{\varvec{B}}})^{-1}Q]_k\\ \sum _{k=1}^n(k-1)[(-\varSigma _{{\varvec{B}}})^{-1}Q]_k\\ \sum _{k=1}^n[(-\varSigma _{{\varvec{B}}})^{-1}Q]_k\end{pmatrix}\nonumber \\&+\frac{\beta \rho \bar{F}(\sigma +\mu )}{\mu (\rho \bar{F}+\sigma +2\mu )}\begin{pmatrix}\sum _{k=1}^n(n-k)[(-\varSigma _{{\varvec{B}}})^{-1}q]_k\\ \sum _{k=1}^n(k-1)[(-\varSigma _{{\varvec{B}}})^{-1}q]_k\\ \sum _{k=1}^n[(-\varSigma _{{\varvec{B}}})^{-1}q]_k\end{pmatrix}, \end{aligned}$$

and the third column is

57 $$\begin{aligned} \begin{pmatrix}k_{AC}\\ k_{{\varvec{B}}C}\\ k_{CC}\end{pmatrix}=\frac{\beta \rho \bar{F}(\sigma +\mu )}{\mu (\rho \bar{F}+\sigma +2\mu )}\begin{pmatrix}\sum _{k=1}^n(n-k)[(-\varSigma _{{\varvec{B}}})^{-1}q]_k\\ \sum _{k=1}^n(k-1)[(-\varSigma _{{\varvec{B}}})^{-1}q]_k\\ \sum _{k=1}^n[(-\varSigma _{{\varvec{B}}})^{-1}q]_k\end{pmatrix}. \end{aligned}$$

Note that the elements $k_{xy}$, $x,y=A,{\varvec{B}},C$ involve the vectors $(-\varSigma _{{\varvec{B}}})^{-1}q$ and $(-\varSigma _{{\varvec{B}}})^{-1}Q$.

We can simplify the sums $\sum _{k=1}^n[(-\varSigma _{{\varvec{B}}})^{-1}q]_k$ and $\sum _{k=1}^n[(-\varSigma _{{\varvec{B}}})^{-1}Q]_k$. Note that

$$\begin{aligned} \sum _{k=1}^n[(-\varSigma _{{\varvec{B}}})^{-1}]_{kl}=\frac{1}{\beta +\sigma +2\mu }, \end{aligned}$$

since $(-\varSigma _{{\varvec{B}}})_{kl}$ is the mean time to spent in state $k$ when starting life in state $l$, by summing over all possible states $k\in {\varvec{B}}$, we obtain the mean time to spent in ${\varvec{B}}$ when starting in some state $l\in {\varvec{B}}$ (this is of course equal to 1 over the rate of leaving ${\varvec{B}}$). Therefore, for any probability distribution ${{\mathrm{\mathbb {P}}}}$,

$$\begin{aligned} \sum _{k=1}^n[(-\varSigma _{{\varvec{B}}})^{-1}{{\mathrm{\mathbb {P}}}}]_k&=\sum _{l=1}^n\left( \sum _{k=1}^n[(-\varSigma _{{\varvec{B}}})^{-1}]_{kl}\right) {{\mathrm{\mathbb {P}}}}_l\\&=\frac{1}{\beta +\sigma +2\mu }\sum _{l=1}^n{{\mathrm{\mathbb {P}}}}_l\\&=\frac{1}{\beta +\sigma +2\mu }, \end{aligned}$$

where the last equality holds since ${{\mathrm{\mathbb {P}}}}$ is a probability distribution. So

58 $$\begin{aligned} \sum _{k=1}^n[(-\varSigma _{{\varvec{B}}})^{-1}q]_k=\sum _{k=1}^n[(-\varSigma _{{\varvec{B}}})^{-1}Q]_k=\frac{1}{\beta +\sigma +2\mu }, \end{aligned}$$

which we can use to simplify $K$.

Observe that the sum of the first and second row of $K$ is $n-1$ times the third row of $K$, i.e.

$$\begin{aligned} k_{A,y}+k_{{\varvec{B}},y}=(n-1)\cdot k_{C,y}, \end{aligned}$$

and the third column is a multiple of the first column, i.e.

$$\begin{aligned} k_{x,C}=\frac{\sigma +\mu }{\sigma +2\mu }k_{x,A}. \end{aligned}$$

So we find that, of the three eigenvalues that $K$ has, at least one equals zero. Moreover, using (58),

$$\begin{aligned} k_{CA}&=\frac{\beta \rho F(\sigma +2\mu )}{\mu (\beta +\sigma +2\mu )(\rho F+\sigma +2\mu )}, \end{aligned}$$

and

$$\begin{aligned} k_{C{\varvec{B}}}&=\frac{\beta }{\beta +\sigma +2\mu }\left( 1+\frac{\rho F(\sigma +\mu )}{\mu (\rho F+\sigma +2\mu )}\right) \!. \end{aligned}$$

To find the dominant eigenvalue of $K$ we can therefore reduce $K$ to the $2\times 2$ matrix $\tilde{K}$:

59 $$\begin{aligned} \tilde{K}&=\begin{pmatrix}k_{AA}+\frac{\sigma +\mu }{\sigma +2\mu }k_{CA}&{} k_{A{\varvec{B}}}+\frac{\sigma +\mu }{\sigma +2\mu }k_{C{\varvec{B}}}\\ k_{{\varvec{B}}A}&{}k_{{\varvec{B}}{\varvec{B}}}\end{pmatrix}\nonumber \\&=\begin{pmatrix}k_{AA}+\frac{\beta \rho F(\sigma +\mu )}{\mu (\beta +\sigma +2\mu )(\rho F+\sigma +2\mu )}&{}k_{A{\varvec{B}}}+\frac{\beta (\rho F+\mu )(\sigma +\mu )}{\mu (\beta +\sigma +2\mu )(\rho F+\sigma +2\mu )}\\ k_{{\varvec{B}} A}&{} k_{{\varvec{B}} {\varvec{B}}}\end{pmatrix}. \end{aligned}$$

Note that

60 $$\begin{aligned} \sum _k(n-k)[-\varSigma _{{\varvec{B}}}^{-1}{{\mathrm{\mathbb {P}}}}]_k&=(n-1)\sum _k[-\varSigma _{{\varvec{B}}}^{-1}{{\mathrm{\mathbb {P}}}}]_k-\sum _k(k-1)[-\varSigma _{{\varvec{B}}}^{-1}{{\mathrm{\mathbb {P}}}}]_k\nonumber \\&=\frac{n-1}{\beta +\sigma +2\mu }-\sum _k(k-1)[-\varSigma _{{\varvec{B}}}^{-1}{{\mathrm{\mathbb {P}}}}]_k, \end{aligned}$$

where ${{\mathrm{\mathbb {P}}}}$ is the probability distribution $q$ or $Q$, and we have used (58) in the second equality. Therefore, the only ingredients left in order to arrive at a completely explicit expression for the dominant eigenvalue $R_0$ of the $2\times 2$ matrix $\tilde{K}$ are explicit expressions for the sums $\sum _{k=1}^n(k-1)[(-\varSigma _{{\varvec{B}}})^{-1}q]_k$ and $\sum _{k=1}^n(k-1)[(-\varSigma _{{\varvec{B}}})^{-1}Q]_k$.

In the remainder of this appendix we show that

61 $$\begin{aligned} \sum _{k}(k-1)[-\varSigma _{{\varvec{B}}}^{-1}q]_k=\frac{(n-1)\rho \bar{F}}{(\rho \bar{F}+\sigma +\mu )(\beta +\sigma +2\mu )}-\frac{(n-1)Y}{\rho F+2\sigma +3\mu +\beta }, \end{aligned}$$

and

62 $$\begin{aligned} \sum _k(k-1)[-\varSigma _{{\varvec{B}}}^{-1}Q]_k&= \frac{(n-1)\rho \bar{F}}{(\rho \bar{F}+\sigma +\mu )(\beta +\sigma +2\mu )}\nonumber \\&-\frac{(n-1)Y(\sigma +2\mu )}{(\rho \bar{F}+2\sigma +3\mu )(\beta +\rho \bar{F}+2\sigma +3\mu )}, \end{aligned}$$

where

63 $$\begin{aligned} Y=\frac{\rho \bar{F}\mu (\rho \bar{F}+2\sigma +3\mu )}{(\sigma +2\mu )(\rho \bar{F}+\sigma +\mu )(2\rho \bar{F}+2\sigma +3\mu )}. \end{aligned}$$

We obtain an explicit expression for $R_0$ by using (60) and plugging (61) and (62) (together with (63)) into (55) and (56). Next, plug these into (59) and use that the dominant eigenvalue of a $2\times 2$ matrix is

$$\begin{aligned} \frac{\text {tr}+\sqrt{\text {tr}^2-4 \text { det}}}{2} \end{aligned}$$

(where tr and det denote the trace and determinant of $\tilde{K}$, respectively).

Finally, in the remainder of this appendix we will show (61) and (62) by straightforward computations that we divide up in four lemma’s (for the first of these we only sketch the proof).

We need the following ingredients. Let $M$ be the matrix corresponding to the state transitions between the states $B_k$, $k=1,\ldots ,n$; see Fig. 13. Then

$$\begin{aligned} \varSigma _{{\varvec{B}}}=M-(\beta +\sigma +2\mu )I. \end{aligned}$$

Furthermore, $e^{tM}$ denotes the fundamental solution of

64 $$\begin{aligned} \frac{d\pi }{dt}=M\pi . \end{aligned}$$

We also use the relationship between $q$ and $Q$:

65 $$\begin{aligned} Q=(\sigma +2\mu )\int _0^\infty e^{-(\sigma +2\mu )t}e^{tM}qdt \end{aligned}$$

(see proof Leung et al. 2012, Lemma 2).

We need the probability distribution $q$ for $n=2$: $q^{(2)}=(q_1^{(2)},q_2^{(2)})^t$ where the superscript $(2)$ is to distinguish the $n=2$ probabilities from general $n>2$.

66 $$\begin{aligned} \begin{aligned} q_1^{(2)}=\frac{\rho \bar{F}\mu +(\sigma +2\mu )(2\sigma +3\mu )}{(\sigma +2\mu )(2\rho \bar{F}+2\sigma +3\mu )},\\ q_2^{(2)}=\frac{\rho \bar{F}(2\sigma +3\mu )}{(\sigma +2\mu )(2\rho \bar{F}+2\sigma +3\mu )}. \end{aligned} \end{aligned}$$

Finally, we use two probabilities for binding sites. Consider one binding site. Conditioning on the individual staying alive till at least time $t$, $\epsilon _0(t)$ and $\epsilon _1(t)$ denote the probabilities that the binding site is occupied at time $t$, given that, respectively, it was free or occupied at time $t=0$. So $\epsilon _i$, $i=0,1$, satisfies

$$\begin{aligned} \frac{d\epsilon _i}{dt}=\rho \bar{F}\epsilon _i-(\sigma +\mu )(1-\epsilon _i) \end{aligned}$$

with initial conditions $\epsilon _0(0)=0$ and $\epsilon _1(0)=1$. Solving these, we find

67 $$\begin{aligned} \begin{aligned} \epsilon _0(t)&=\frac{\rho \bar{F}}{\rho \bar{F}+\sigma +\mu }-\frac{\rho \bar{F}}{\rho \bar{F}+\sigma +\mu }e^{-(\rho \bar{F}+\sigma +\mu )t},\\ \epsilon _1(t)&=\frac{\rho \bar{F}}{\rho \bar{F}+\sigma +\mu }+\frac{\sigma +\mu }{\rho \bar{F}+\sigma +\mu }e^{-(\rho \bar{F}+\sigma +\mu )t}. \end{aligned} \end{aligned}$$

### **Lemma 6**

68 $$\begin{aligned} \sum _{k=2}^n(k-1)(e^{tM})_{kj}=(n-j)\epsilon _0(t)+(j-1)\epsilon _1(t), \end{aligned}$$

### *Sketch of proof*

Consider a randomly chosen partnership between two individuals $u$ and $v$. Then $(e^{tM})_{kj}$ is the probability for $u$ to be in state $k$ given that it starts life in $j$ (here: ‘life starts’ at the moment $uv$ is formed). Then $\sum _{k=2}^n(k-1)(e^{tM})_{kj}$ is the expected number of partners of $u$, minus partner $v$, at time $t$ given that $u$ started life in $j$. Conditioning on the existence of $uv$, the other $n-1$ binding sites of $u$ behave independently of one another.

Since $u$ starts life in state $j$, there are $j-1$ binding sites that have probability $\epsilon _1(t)$ to be occupied at time $t$ and $n-j$ binding sites that have probability $\epsilon _0(t)$ to be occupied at time $t$. Therefore, the expected number of occupied binding sites of $u$ minus the binding site occupied by $v$ at time $t$ is exactly the right hand side of (68).   $\square$

We now consider the expected number of ‘other’ partners of an individual that just acquired a new partner in the following lemma.

### **Lemma 7**

$$\begin{aligned} \sum _{k=1}^n(k-1)q_k=(n-1)q_2^{(2)}, \end{aligned}$$

with $q_2^{(2)}$ given by (66).

### *Proof*

The probability $q_k$ is given by (7) with

$$\begin{aligned} P_{k-1}=\left( {\begin{array}{c}n\\ k-1\end{array}}\right) \int _0^\infty \mu e^{-\mu a}(1-\epsilon _0(a))^{n-k+1}\epsilon _0(a)^{k-1}da. \end{aligned}$$

Then

$$\begin{aligned}&(k-1)(n-k+1)P_{k-1}\\&\quad =\frac{n!}{(k-2)!(n-k)!}\int _0^\infty \mu e^{-\mu a}(1-\epsilon _0(a))\epsilon _0(a)(1-\epsilon _0(a))^{n-k}\epsilon _0(a)^{k-2}da\\&\quad =n(n-1)\int _0^\infty \mu e^{-\mu a}(1-\epsilon _0(a))\epsilon _0(a)\left( {\begin{array}{c}n-2\\ k-2\end{array}}\right) (1-\epsilon _0(a))^{n-k}\epsilon _0(a)^{k-2}da \end{aligned}$$

If we now take the sum $\sum _k(k-1)q_k$, then we obtain

$$\begin{aligned} \sum _{k=2}^nq_k&=\frac{n(n-1)}{n\bar{F}}\int _0^\infty \mu e^{-\mu a}(1-\epsilon _0(a))\epsilon _0(a)da\\&=\frac{n-1}{2\bar{F}}P_1^{(2)}\\&=(n-1)q_2^{(2)}, \end{aligned}$$

which we wanted to show. $\square$

### **Lemma 8**

$$\begin{aligned} \sum _{k=2}^n(k-1)(e^{tM}q)_k=(n-1)\left( \frac{\rho F}{\rho F+\sigma +\mu }-Y e^{-t(\rho \bar{F}+\sigma +\mu )}\right) , \end{aligned}$$

where $Y$ is the positive constant (63).

### *Proof*

We combine Lemmas 6 and 7.

$$\begin{aligned} \sum _{k=2}^n(k-1)(e^{tM}q)_k&=\sum _{k=2}^n(k-1)\sum _{j=1}^n(e^{tM})_{kj}q_j\\&=\sum _{j=1}^nq_j\sum _{k=2}^n(k-1)(e^{tM})_{kj}\\ \text {(Lemma 6)}&=\sum _{j=1}^nq_j\left( (n-j)\epsilon _0(t)+(j-1)\epsilon _1(t)\right) \\ \text {(Lemma 7)}&=(n-1)(1-q_2^{(2)})\epsilon _0(t)+(n-1)q_2^{(2)}\epsilon _1(t)\\&=(n-1)\left( q_1^{(2)}\epsilon _0(t)+q^{(2)}_2\epsilon _1(t)\right) . \end{aligned}$$

Finally, one can use (66) and (67) in the last step to arrive at the explicit expression. $\square$

### **Lemma 9**

The equalities (61) and (62) hold.

### *Proof*

Putting all the pieces together, we obtain

$$\begin{aligned} \sum _{k=2}^n(k-1)[-\varSigma _{{\varvec{B}}}^{-1}q]_k&=\sum _k(k-1)\left( \int _0^\infty e^{-(\beta +\sigma +2\mu )t}e^{tM}qdt\right) _k\\&=\sum _k(k-1)\int _0^\infty e^{-(\beta +\sigma +2\mu )t}(e^{tM}q)_kdt\\&=\int _0^\infty e^{-(\beta +\sigma +2\mu )t}\sum _k(k-1)(e^{tM}q)_kdt\\ \text {(Lemma 8) }&=\int _0^\infty e^{-(\beta +\sigma {+}2\mu )t} (n{-}1)\left( \frac{\rho F}{\rho F+\sigma +\mu }-Y e^{-t(\rho \bar{F}+\sigma +\mu )}\right) dt\\&=\frac{(n-1)\rho \bar{F}}{(\rho \bar{F}+\sigma +\mu )(\beta +\sigma +2\mu )}-\frac{(n-1)Y}{\rho F+2\sigma +3\mu +\beta }. \end{aligned}$$

and for the sum involving $Q$, we use (65), and then find

$$\begin{aligned}&\sum _{k=2}^n(k-1)[-\varSigma _{{\varvec{B}}}^{-1}Q]_k\\&=\sum _k(k-1)\left( \int _0^\infty e^{-(\beta +\sigma +2\mu )t}e^{tM}Qdt\right) _k\\&=\sum _k(k-1)\left( \int _0^\infty e^{-(\beta +\sigma +2\mu )t}e^{tM}(\sigma +2\mu )\int _0^\infty e^{-(\sigma +2\mu )\tau }e^{\tau M}qd\tau dt\right) _k\\&= (\sigma +2\mu )\sum _k(k-1)\left( \int _0^\infty \int _0^\infty e^{-(\beta +\sigma +2\mu )t}e^{-(\sigma +2\mu )\tau }e^{(t+\tau )M}qd\tau dt\right) _k\\&=(\sigma +2\mu )\int _0^\infty \int _0^\infty e^{-(\beta +\sigma +2\mu )t}e^{-(\sigma +2\mu )\tau }\sum _k(k-1)\left( e^{(t+\tau )M}q\right) _kd\tau dt\\ \text {(Lemma~8)}&=(\sigma +2\mu )\int _0^\infty \int _0^\infty \left[ e^{-(\beta +\sigma +2\mu )t}e^{-(\sigma +2\mu )\tau }\right. \\&\left. (n-1)\left( \frac{\rho F}{\rho F+\sigma +\mu }-Y e^{-(t+\tau )(\rho \bar{F}+\sigma +\mu )}\right) \right] d\tau dt\\&= \frac{(n-1)\rho \bar{F}}{(\rho \bar{F}+\sigma +\mu )(\beta +\sigma +2\mu )}-\frac{(n-1)Y(\sigma +2\mu )}{(\rho \bar{F}+2\sigma +3\mu )(\beta +\rho \bar{F}+2\sigma +3\mu )}. \end{aligned}$$

$$\square $$

Finally, we note that we can use the method described in this appendix to find explicit expressions for the matrix entries of the $3\times 3$ matrix $M_r$, that are characterized by identity (47). Note that we can find expressions for the $T(rI-\varSigma )^{-1}\psi _x$, $x=A,{\varvec{B}},C$ by simply replacing $-\varSigma _{{\varvec{B}}}$ by $rI-\varSigma _{{\varvec{B}}}$ in the calculations in this appendix. This allows us to characterize $(m_r)_{xy}$. We refrain from elaborating the details.

## Appendix D: Mean field at distance one—bounds for $R_0$

As explained in the main text, the mean field at distance one assumption is a moment closure approximation as we ignore certain correlations between the states of two individuals in a partnership. One may wonder how well ‘the real $R_0$’ (presuming it can be defined, when no assumption is made about the degree distribution of the partners of an individual at epi-birth) is approximated by $R_0$ as derived under the mean field at distance one assumption. Note that here we focus on the mean field at distance one assumption in the linearised system only.

In this appendix we provide lower- and upper bounds for ‘the real $R_0$’ for the case $n=2$ with numerical values presented in Fig. 14.

Consider a randomly chosen partnership with partners $u$ and $v$. Assume that the partnership is formed at time $t=0$. Then, as long as we condition on the existence of partnership $uv$, the states of $u$ and $v$ are independent from one another at time $t=s$ (as also explained in Appendix B). The probability that $u$ is in state 1 or 2 at time $s$ is given by the probability distribution

$$\begin{aligned} e^{sM}q, \end{aligned}$$

$$s\ge 0$$, where

$$\begin{aligned} M=\begin{pmatrix}-\rho \bar{F}&{}\sigma +\mu \\ \rho \bar{F}&{}-(\sigma +\mu ) \end{pmatrix}. \end{aligned}$$

For $s=0$, this yields initial condition $q$, for $s\rightarrow \infty$ we obtain probability distribution

$$\begin{aligned} q^\infty =\begin{pmatrix}\frac{\sigma +\mu }{\rho \bar{F}+\sigma +\mu }\\ \frac{\rho \bar{F}}{\rho \bar{F}+\sigma +\mu }\end{pmatrix}. \end{aligned}$$

Then

$$\begin{aligned} q_2\le P(v\text { has degree 2 at time }s\mid u\text { has degree }k\text { at time }s)=\left( e^{sM}q\right) _2\le q_2^\infty \end{aligned}$$

(note that the equality holds since the degrees of $u$ and $v$ are independent of one another as long as we specify the duration of the partnership $uv$). Therefore

69 $$\begin{aligned} q_2\le P(v\text { has degree 2}\mid u\text { has degree }k)\le q_2^\infty , \end{aligned}$$

$k=1,2$ (recall that we condition on the existence of partnership $uv$). Under the mean field at distance one assumption, we say that

$$\begin{aligned} P(v\text { has degree 2}\mid u\text { has degree }k)=Q_2. \end{aligned}$$

As we will explain now, (69) provides us with a bandwidth for ‘the real $R_0$’ (in which the mean field at distance one $R_0$ also falls).

The mean field at distance one assumption manifests itself in the distribution $Q$, which plays a role in elements $k_{A{\varvec{B}}}$ and $k_{{\varvec{B}}{\varvec{B}}}$. For $n=2$, we find $\sum _{k=1}^n(n-k)[-\varSigma _{{\varvec{B}}}^{-1}Q]_k=[-\varSigma _{{\varvec{B}}}^{-1}Q]_1$ and $\sum _{k=1}^n(k-1)[-\varSigma _{{\varvec{B}}}^{-1}Q]_k=[-\varSigma _{{\varvec{B}}}^{-1}Q]_2$.

If we replace $Q$ by a probability distribution ${{\mathrm{\mathbb {P}}}}$ in (59) (and keep all other elements equal) then we deal with $[-\varSigma _{{\varvec{B}}}^{-1}{{\mathrm{\mathbb {P}}}}]_j$, $j=1,2$. Using (58) we find that $[-\varSigma _{{\varvec{B}}}^{-1}{{\mathrm{\mathbb {P}}}}]_1+[-\varSigma _{{\varvec{B}}}^{-1}{{\mathrm{\mathbb {P}}}}]_2=1/(\beta +\sigma +2\mu )$ holds so we can eliminate $[-\varSigma _{{\varvec{B}}}^{-1}{{\mathrm{\mathbb {P}}}}]_1$. (All other elements in (59) do not concern the mean field at distance one assumption, so for any assumption on the degree distribution of the partners of an individual at epi-birth, these will be the same.) We can then express the dominant eigenvalue $\lambda$ of $\tilde{K}$ as a function of $[-\varSigma _{{\varvec{B}}}^{-1}{{\mathrm{\mathbb {P}}}}]_2$ using the explicit formula for the dominant eigenvalue of a $2\times 2$ matrix. This is then a monotonically increasing function of $[-\varSigma _{{\varvec{B}}}^{-1}{{\mathrm{\mathbb {P}}}}]_2$.

Furthermore, one can check that

$$\begin{aligned}{}[-\varSigma _{{\varvec{B}}}^{-1}{{\mathrm{\mathbb {P}}}}]_2&=[-\varSigma _{{\varvec{B}}}^{-1}]_{21}(1-{{\mathrm{\mathbb {P}}}}_2)+[-\varSigma _{{\varvec{B}}}^{-1}]_{22}{{\mathrm{\mathbb {P}}}}_2\\&=[-\varSigma _{{\varvec{B}}}^{-1}]_{21}+([-\varSigma _{{\varvec{B}}}^{-1}]_{22}-[-\varSigma _{{\varvec{B}}}^{-1}]_{21}){{\mathrm{\mathbb {P}}}}_2, \end{aligned}$$

where $[-\varSigma _{{\varvec{B}}}^{-1}]_{22}\ge [-\varSigma _{{\varvec{B}}}^{-1}]_{21}$ (note that $\varSigma _{{\varvec{B}}}=M-(\beta +\sigma +2\mu )I$), so $[-\varSigma _{{\varvec{B}}}^{-1}{{\mathrm{\mathbb {P}}}}]_2$ is a monotonically increasing function of ${{\mathrm{\mathbb {P}}}}_2$. Using (69) we then find

$$\begin{aligned}{}[-\varSigma _{{\varvec{B}}}^{-1}q]_2\le [-\varSigma _{{\varvec{B}}}^{-1}Q]_2 \le [-\varSigma _{{\varvec{B}}}^{-1}q^\infty ]_2, \end{aligned}$$

and we find a lower (upper) bound by replacing ${{\mathrm{\mathbb {P}}}}$ by $q$ ($q^\infty$) for ‘the real $R_0$’ which we can compare with the dominant eigenvalue $R_0$ of (59). We evaluate this numerically in Fig. 14 to get some indication of how well the mean field at distance assumption performs.

We believe that this can be generalized to obtain a bandwidth for $R_0$ for $n>2$ by considering expected values but we have not elaborated the details.

## Appendix E: The dominant eigenvalue of $K$ equals the dominant eigenvalue of $K^{\text {ind}}$

We have defined $R_0$ on the binding-site level as the dominant eigenvalue of a NGM $K$. In this section we prove that $K$ and $K^\text {ind}$, the NGM on the individual level, have the same dominant eigenvalue $R_0$. Therefore, $R_0$ has an interpretation on both the binding site and the individual level.

### **Lemma 10**

$K^\text {ind}$ and $K$ have the same dominant eigenvalue $R_0$.

### *Proof*

With the $3\times n$ matrix $G$ given by

70 $$\begin{aligned} G=\left( \begin{array}{ccccc}n-1 &{} n-2 &{} \cdots &{} 1 &{} 0\\ 0&{} 1 &{}\cdots &{} n-2 &{} n-1\\ 1 &{} 1&{} \cdots &{}1 &{}1 \end{array}\right) \end{aligned}$$

the identity

71 $$\begin{aligned} GK^\text {ind}=K G \end{aligned}$$

holds. Since $T\ge 0$, $T\ne 0$, $-\varSigma ^{-1}>>0$, $\psi , \phi _i\ge 0$, $\psi , \phi \ne 0$, we know that $(k^{\text {ind}})_{xy}>0$ and $k_{ij}>0$ for all $x,y$. Therefore $K^{\text {ind}}$ and $K$ are primitive matrices.

Now suppose that $v$ is the eigenvector corresponding to eigenvalue $R_0$, then $K^\text {ind}v=R_0v$ implies that

$$\begin{aligned} K(Gv)=R_0(Gv). \end{aligned}$$

Since $v$ can be chosen as a strictly positive vector (as it belongs to the dominant eigenvalue of a primitive matrix) and $G$ is non-negative and non-zero, $Gv$ is also strictly positive. Therefore, $Gv$ is the (strictly positive) eigenvector corresponding to the eigenvalue $R_0$ of $K$. Since $K$ is primitive, it must also be the dominant eigenvalue of $K$.

On the other hand, suppose $w$ is the eigenvector corresponding to the dominant eigenvalue $\lambda$ of $K^t$ (where $^t$ denotes the transpose of $K$), so $K^tw=\lambda w$. Then we can choose $w$ strictly positive, as $K$ is primitive. Then

$$\begin{aligned} (K^\text {ind})^t(G^tw)=\lambda (G^tw). \end{aligned}$$

So we see that $\lambda$ is an eigenvalue of $(K^\text {ind})^t$ (and therefore also of $K^\text {ind}$) with strictly positive eigenvector $G^tw$. Therefore, $\lambda$ is the dominant eigenvalue of $K^\text {ind}$, i.e. $\lambda =R_0$. $\square$

## Appendix F: Characterizing the matrix elements $(k^{\text {ind}})_{ij}$

The matrix elements $(k^\text {ind})_{ij}$ of the matrix $K^\text {ind}$ are uniquely characterized in (37). However, as in the case with $K$, we can give more explicit expressions for $(k^\text {ind})_{ij}$ using the interpretation.

Indeed, $(k^{\text {ind}})_{ij}$ can be interpreted as the expected number of secondary cases in state i caused by one individual in state $j$, where i and $j$ are of the form $(+,m,1)$, $m=0,\ldots ,n-1$. A newly infected individual in state $j=(+,m,1)$ has $m$ susceptible partners, where each of these partners is in state $k=(-,l,1)$ with probability $Q_l$ (as a consequence of the mean field at distance one assumption). The probability that an individual in state $k$ gets infected and has state-at-epi-birth $i=(+,m',1)$ is $[\beta (-\varSigma _{{\varvec{B}}})^{-1}]_{ik}$. On top of the secondary cases caused by infecting existing partners, the newly infected individual can also cause secondary cases among the partners that it acquires after epi-birth. A newly acquired partner is in state $k=(-,l,1)$ with probability $q_l$, and the probability that this individual gets infected and has state-at-epi-birth i is again $[\beta (-\varSigma _{{\varvec{B}}})^{-1}]_{ik}$. The expected additional lifetime number of partners of an individual in state $j=(+,m,1)$ is $n-m-1$ times the expected lifetime number of partners of a free binding site plus $m+1$ times the expected additional lifetime number of partners of an occupied binding site, where the expected lifetime number of partners of a free binding site is

$$\begin{aligned} \frac{\rho \bar{F}(\sigma +2\mu )}{\mu (\rho \bar{F}+\sigma +2\mu )}, \end{aligned}$$

while the expected additional lifetime number of partners of an occupied binding site is

$$\begin{aligned} \frac{\sigma +\mu }{\sigma +2\mu }\frac{\rho \bar{F}(\sigma +2\mu )}{\mu (\rho \bar{F}+\sigma +2\mu )}=\frac{\rho \bar{F}(\sigma +\mu )}{\mu (\rho \bar{F}+\sigma +2\mu )}. \end{aligned}$$

Using this interpretation of the matrix elements $(k^{\text {ind}})_{ij}$ we find

72 $$\begin{aligned} K^{\text {ind}}=\beta (-\varSigma _{{\varvec{B}}})^{-1}(Qr^t+qs^t), \end{aligned}$$

with $s=(s_k)$ and $r=(r_k)$ are $n$-dimensional vectors, where $r$ is the second row of the matrix $G$ defined in (70), i.e.

$$\begin{aligned} r_k=k-1, \end{aligned}$$

$$k=1,\ldots ,n$$, and

$$\begin{aligned} s_k=\left( (n-k)+k\frac{\sigma +\mu }{\sigma +2\mu }\right) \frac{\rho \bar{F}(\sigma +2\mu )}{\mu (\rho \bar{F}+\sigma +2\mu )}. \end{aligned}$$

In order to prove that $K^{\text {ind}}$, defined by (37), satisfies (72), we use results from Appendix C. First of all, $\phi _j$ can be written as

$$\begin{aligned} \phi _j=(n-j)\psi _A+(j-1)\psi _{{\varvec{B}}}+\psi _C. \end{aligned}$$

Therefore,

$$\begin{aligned} T(-\varSigma )^{-1}\phi _j&=(n-j)T(-\varSigma )^{-1}\psi _A+(j-1)T(-\varSigma )^{-1}\psi _{{\varvec{B}}}+T(-\varSigma )^{-1}\psi _C\\&=(n-j)\sum _{x}k_{xA}\psi _x+(j-1)\sum _{x}k_{x,{\varvec{B}}}\psi _x+\sum _{x}k_{xC}\psi _i\\&=\left( (n-j)k_{AA}+(j-1)k_{A{\varvec{B}}}+k_{AC}\right) \psi _A\\&+\left( (n-j)k_{{\varvec{B}}A}+(j-1)k_{{\varvec{B}}{\varvec{B}}}+k_{{\varvec{B}}C}\right) \psi _{{\varvec{B}}}\\&+\left( (n-j)k_{CA}+(j-1)k_{C{\varvec{B}}}+k_{CC}\right) \psi _C. \end{aligned}$$

Now, we can expand this using the characterization of the $k_{xy}$ of Appendix C. The coefficient of $\psi _C$ is

$$\begin{aligned}&(n-j)k_{CA}+(j-1)k_{C{\varvec{B}}}+k_{CC}\\&\quad =(n-j)\frac{\beta \rho \bar{F}(\sigma +2\mu )}{\mu (\rho \bar{F}+\sigma +2\mu )}\sum _{i=1}^n[(-\varSigma _{{\varvec{B}}})^{-1}q]_i+(j-1)\sum _{i=1}^n[(-\varSigma _{{\varvec{B}}})^{-1}Q]_i\\&\quad +(j-1)\frac{\beta \rho \bar{F}(\sigma +\mu )}{\mu (\rho \bar{F}+\sigma +2\mu )}\sum _{i=1}^n[(-\varSigma _{{\varvec{B}}})^{-1}q]_i\\&\quad +\frac{\beta \rho \bar{F}(\sigma +\mu )}{\mu (\rho \bar{F}+\sigma +2\mu )}\sum _{i=1}^n[(-\varSigma _{{\varvec{B}}})^{-1}q]_i\\&\quad =\beta \left( (n-j)\frac{\rho \bar{F}(\sigma +2\mu )}{\mu (\rho \bar{F}+\sigma +2\mu )}+j\frac{\rho \bar{F}(\sigma +\mu )}{\mu (\rho \bar{F}+\sigma +2\mu )}\right) \sum _{i=1}^n[(-\varSigma _{{\varvec{B}}})^{-1}q]_i\\&\quad +\beta (j-1)\sum _{i=1}^n[(-\varSigma _{{\varvec{B}}})^{-1}Q]_i\\&\quad =\sum _{i=1}^n\beta \left( [(-\varSigma _{{\varvec{B}}})^{-1}q]_is_j+[(-\varSigma _{{\varvec{B}}})^{-1}Q]_ir_j\right) \\&\quad =\sum _{i=1}^n\beta \left[ (-\varSigma _{{\varvec{B}}})^{-1}(qs^t+Qr^t)\right] _{ij}. \end{aligned}$$

On the other hand,

$$\begin{aligned} T(-\varSigma )^{-1}\phi _j&=\sum _{i=1}^n(k^{\text {ind}})_{ij}\phi _i\\&=\sum _{i=1}^n(n-i)(k^{\text {ind}})_{ij}\psi _A+\sum _{i=1}^n(i-1)(k^{\text {ind}})_{ij}\psi _{{\varvec{B}}}+\sum _{i=1}^n(k^{\text {ind}})_{ij}\psi _C. \end{aligned}$$

Therefore

$$\begin{aligned} (k^{\text {ind}})_{ij}&=\beta \left[ (-\varSigma _{{\varvec{B}}})^{-1}(qs^t+Qr^t)\right] _{ij}\!. \end{aligned}$$
